# Impact of RNA degradation on fusion detection by RNA-seq

**DOI:** 10.1186/s12864-016-3161-9

**Published:** 2016-10-20

**Authors:** Jaime I. Davila, Numrah M. Fadra, Xiaoke Wang, Amber M. McDonald, Asha A. Nair, Barbara, R. Crusan, Xianglin Wu, Joseph H. Blommel, Jin Jen, Kandelaria M. Rumilla, Robert B. Jenkins, Umut Aypar, Eric W. Klee, Benjamin R. Kipp, Kevin C. Halling

**Affiliations:** 1 Department of Health Science Research, Mayo Clinic, Rochester, MN 55905 USA; 2 Department of Laboratory Medicine and Pathology, Mayo Clinic, Rochester, MN 55905 USA; 3 Genome Analysis Core, Medical Genome Facility, Center for Individualized Medicine, Mayo Clinic, Rochester, MN 55905 USA

**Keywords:** RNA-seq, Fusion detection, RNA degradation, Poly-A pulldown

## Abstract

**Background:**

RNA-seq is a well-established method for studying the transcriptome. Popular methods for library preparation in RNA-seq such as Illumina TruSeq® RNA v2 kit use a poly-A pulldown strategy. Such methods can cause loss of coverage at the 5′ end of genes, impacting the ability to detect fusions when used on degraded samples. The goal of this study was to quantify the effects RNA degradation has on fusion detection when using poly-A selected mRNA and to identify the variables involved in this process.

**Results:**

Using both artificially and naturally degraded samples, we found that there is a reduced ability to detect fusions as the distance of the breakpoint from the 3′ end of the gene increases. The median transcript coverage decreases exponentially as a function of the distance from the 3′ end and there is a linear relationship between the coverage decay rate and the RNA integrity number (RIN). Based on these findings we developed plots that show the probability of detecting a gene fusion (“sensitivity”) as a function of the distance of the fusion breakpoint from the 3′ end.

**Conclusions:**

This study developed a strategy to assess the impact that RNA degradation has on the ability to detect gene fusions by RNA-seq.

**Electronic supplementary material:**

The online version of this article (doi:10.1186/s12864-016-3161-9) contains supplementary material, which is available to authorized users.

## Background

RNA-seq [1] is a popular method that uses Next Generation Sequencing (NGS) to assess the diversity of the transcriptome. Such method has been used successfully to measure gene expression [2], identify gene fusions [3–5] and detect expressed Single Nucleotide Variants (eSNV) [6, 7] and is increasingly used in the clinical realm [8].

The major steps involved in RNA-seq include 1) library preparation where the RNA is converted into small fragments of cDNA, 2) NGS where those DNA fragments are sequenced and 3) bioinformatics analysis where the fragments are aligned to the reference genome and processed to identify features of biological interest.

The Illumina TruSeq RNA v2 kit is a popular method for next generation sequencing library preparation from mRNA that uses a “poly-A pulldown” which refers to the use of oligo (dT) coated magnetic beads to capture polyadenylated mRNA. Large projects such as The Cancer Genome Atlas (TCGA) [9] and the Genotype-Tissue Expression (GTEx) [10] have used this method extensively. It is already known that when sequencing partially degraded samples, the poly-A pulldown chemistry causes less read coverage at the 5′ end of the gene and results in biases in gene quantification. Because of this, a variety of methods [11–15] have been developed that assess the level of degradation at the sample or transcript level and reduce this bias when performing gene quantification. The earliest method [13] represents the RNA degradation at the transcript level using an exponential model and proposes a method to estimate the isoform expression. Another method [12] argues for the use of the RNA Integrity Number (RIN) as a variable in a linear model to correct for the effect of degradation in gene quantification. 3′ Tag Counting [11] performs gene quantification by considering only reads that occur within a particular distance of the 3′ end. The mRNA integrity number (mRIN) [15] uses a modified Kolmogorov-Smirnov statistic to model the 3′ bias at the transcript level and argues for the use of such metric to exclude samples from the analysis. A similar method, the Transcript Integrity Number (TIN) calculates the entropy of coverage at the transcript level and uses such measure to adjust the gene expression. These methods do not address the effect of degradation in fusion detection which we describe next.

Gene fusions are chimeric transcripts where parts of two known genes are expressed in a single transcript. RNA-seq has been successful in identifying fusions and there are numerous tools with which to do this [4, 16–19]. These tools work by detecting and aggregating reads which either span the fusion junction (usually referred to as spanning reads) or read pairs where each part of the read maps to a different gene (usually referred to as encompassing reads). The sequencing depth in an RNA-seq experiment impacts the number of spanning and encompassing reads, hence the sensitivity of popular fusion detection tools decreases as the total sequencing depth is reduced and reaches a peak at around 15 to 35 million reads [18].

A recent study [20] reports that poly-A pulldown libraries negatively affect fusion detection in degraded samples and argues for the use of an exome capture step during library preparation. Furthermore, it has been shown that methods that avoid the use of poly-A selection such as RiboMinus [11] or capture enrichment [20] do not suffer from 3′ bias. However, given the large amount of publicly available RNA-seq data using poly-A pulldown libraries, we sought to quantify the effects RNA degradation has on fusion detection accuracy when using the Illumina TruSeq method and to characterize the variables involved in this process and their effect on the sensitivity of fusion detection. To do this we utilized RNA isolated from the KU812 tumor cell line [21] and the Universal Human Reference (UHR) RNA tumor samples [22] and experimentally degraded the RNA in these samples to different RNA Integrity Number (RIN) [23] values. We also explored the impact RNA degradation had on fusion detection in normal and tumor specimens with naturally varying levels of RNA degradation.

## Results

### In a degraded chronic myelogenous leukemia (CML) cell line the *BCR-ABL* fusion was not detected while the reciprocal *ABL-BCR* fusion was found

KU812 is a myeloid cell line established from a patient with chronic myelogenous leukemia (CML) [21]. One of the hallmarks of CML is the t (9;22) (q34;q11.2) [24] translocation that results in a *BCR-ABL* fusion [25] which is a driver of the disease. In an attempt to characterize the effect of RNA degradation on the detection of fusions by RNA-seq we artificially degraded RNA isolated from the KU812 cell line to various RIN values (10, 7, 5, and 3) and performed RNA-seq.

**Fig. 1:**
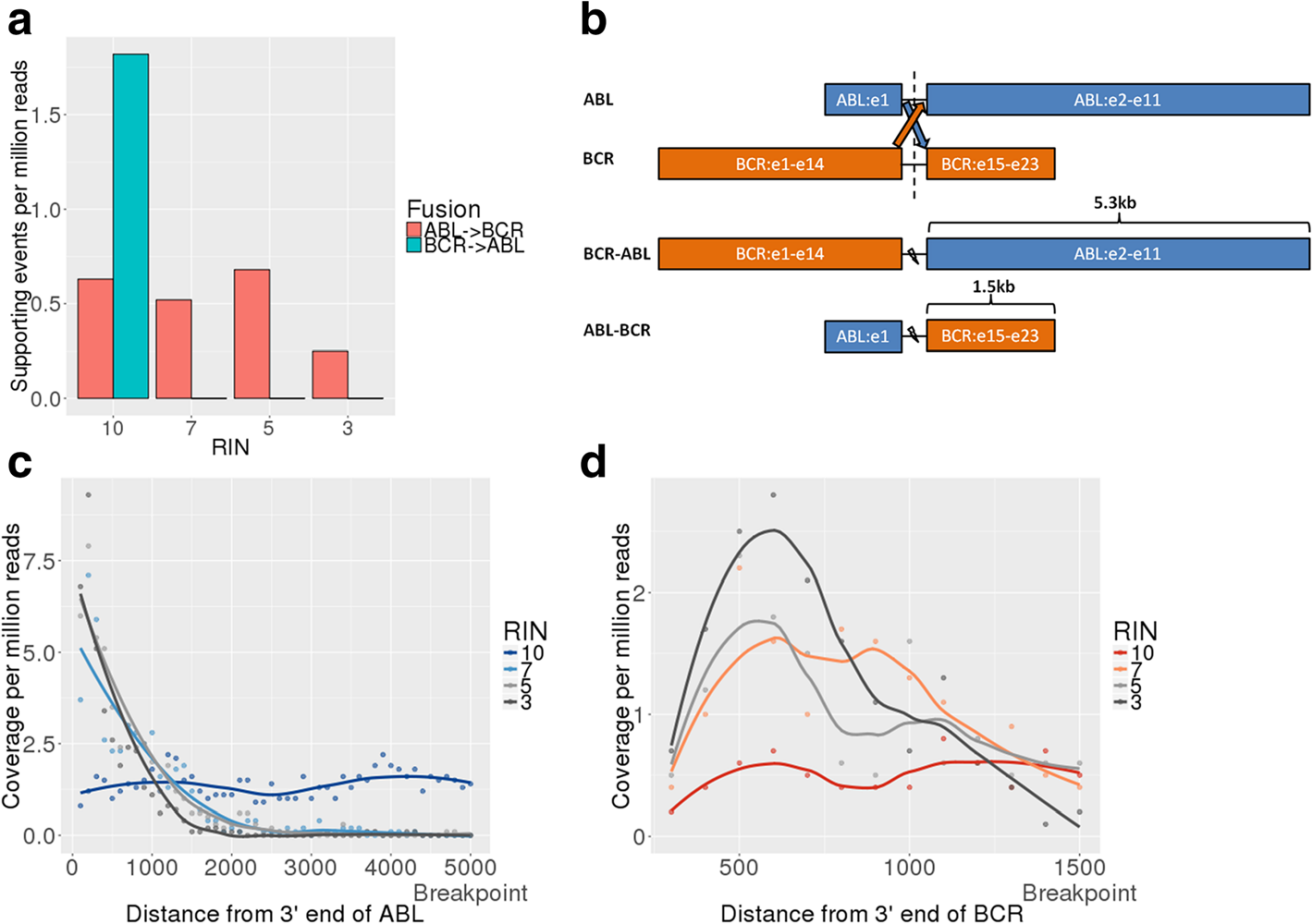
Identification of the *BCR*-*ABL* and *ABL*-*BCR fusions* in chemically degraded RNA from a KU812 cell line using the TruSeq RNA-seq protocol. **a** Supporting reads per million reads for *BCR*-*ABL* and *ABL*-*BCR* at different RIN values (10, 7, 5, 3). **b** Diagram of *ABL* and *BCR* genes and the *BCR*-*ABL* and *ABL*-*BCR* fusion products showing the different distances of each fusion product from the 3′ end. **c** Coverage level per million reads as a function of the distance from the 3′ end for the *ABL*-*BCR* fusion at different levels of degradation. A loess trend line is depicted for each sample. **d** Coverage level per million reads as a function of the distance from the 3′ end for the *BCR*-*ABL* fusion at different levels of degradation. A loess trend line is depicted for each sample

Using the intact RNA (RIN 10) from the KU812 cell line we identified a *BCR*-*ABL* fusion with 1.82 supporting events per million reads (around 27 supporting reads in a sample with 15 million reads) and its reciprocal *ABL*-*BCR* fusion with 0.63 supporting events per million reads (around 9 supporting reads in sample with 15 million reads) (Fig. 1a). In the degraded samples (RIN values 7, 5 and 3) we detected the *ABL*-*BCR* fusion but did not detect the BCR-ABL fusion (Fig. 1a). We noticed that the two fusion products have substantially different distances from the fusion breakpoint to the 3′ end of the transcript (approximately 1.5 kb for *ABL-BCR* and 5.3 kb for *BCR*-*ABL*) (Fig. 1b). We speculated that this might explain the differences in fusion product supporting reads identified for the *ABL-BCR* and *BCR-ABL* fusion at different RIN values.

A plot of the number of reads starting from the 3′end up to the *BCR-ABL* breakpoint (Fig. 1c, Additional file 1: Figure S1a) and *ABL*-*BCR* fusion breakpoint (Fig. 1d, Additional file 1: Figure S1b) reveals that the level of coverage is relatively constant for the sample with a RIN of 10. However, for both fusions RNA degradation caused an increase in read counts at the 3′ end of the transcript, with a subsequent non-linear decrease in read counts as a function of the 3′ distance. The number of reads close to the *ABL-BCR* breakpoint is similar across all levels of degradation (Fig. 1d, Additional file 1: Figure S1b) resulting in the detection of the *ABL-BCR* fusion at all four RIN values (Fig. 1a). In contrast, the number of reads close to the *BCR-ABL* breakpoint drops dramatically and results in the inability to detect such a fusion in the degraded samples (Fig. 1c, Additional file 1: Figure S1a).

### Median coverage decreases exponentially as a function of the distance from the 3′ end

**Fig. 2:**
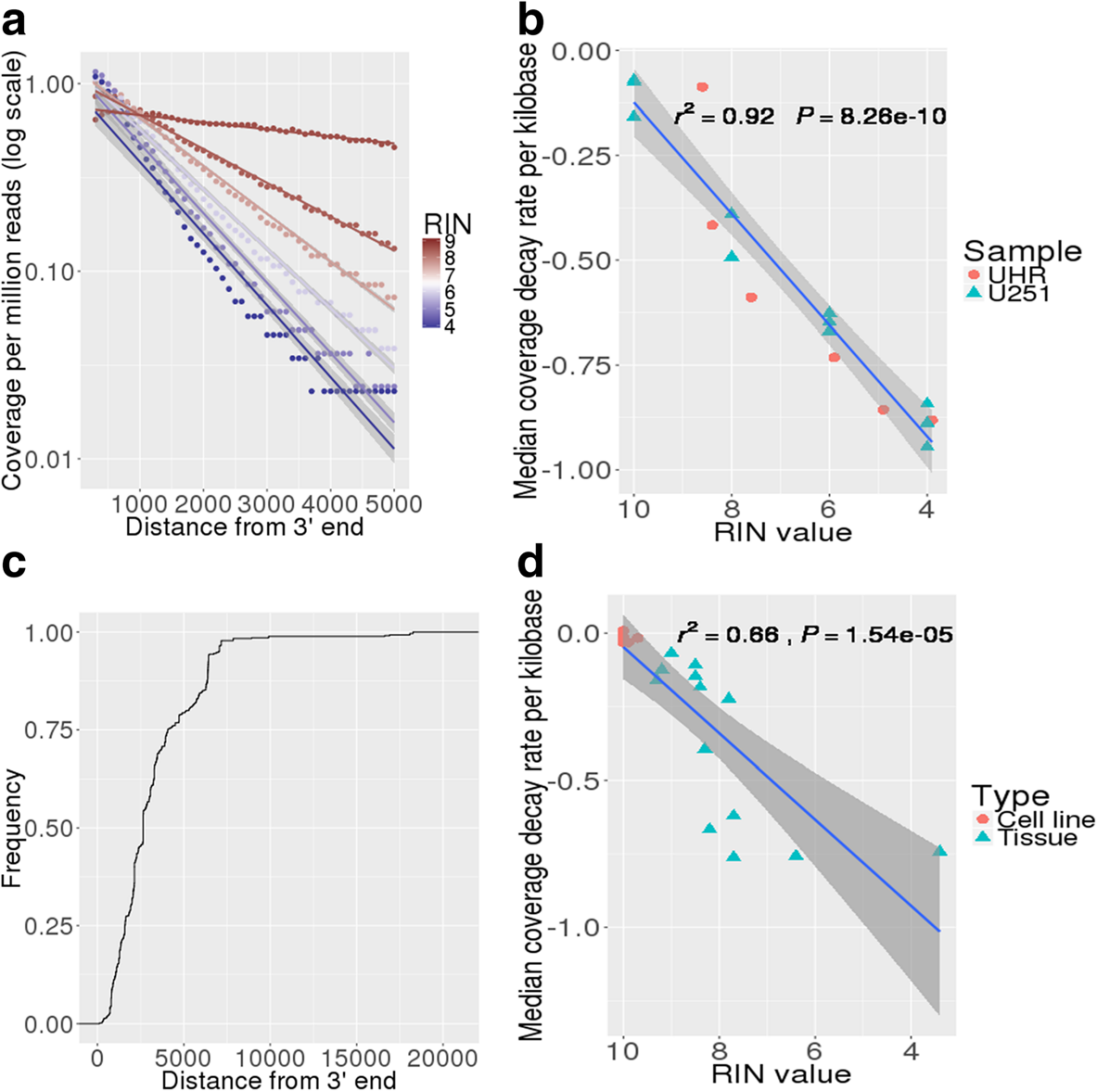
Median coverage profile as a function of distance from 3′ end of mRNA and read decay across all protein-coding genes and genes involved in fusions for samples with different levels of degradation. **a** Median coverage per million reads (in log scale) as function of the distance from the 3′ end for UHR sample at different RIN values (8.6, 8.4, 7.6, 5.9, 4.9 and 3.9). Linear trend lines and 95 % confidence intervals are denoted in *gray*. **b** Median read decay rate per kilobase for all genes as a function of RIN for chemically degraded samples (UHR and U251 cell line). Notice that U251 had replicates. Linear trend line is shown in *blue* and 95 % confidence intervals are shown in *gray*. **c** Cumulative histogram of the distance of gene fusion breakpoints from the 3′ end for fusions in the COSMIC database. **d** Median read decay rate per kilobase for fusion related genes as function of RIN for a set of 20 fresh tissue specimens. Linear trend line is shown in *blue* and 95 % confidence intervals are shown in *gray*

To more accurately characterize the effect RNA degradation has on read coverage of the transcripts we chemically degraded Universal Human Reference RNA (UHR) to six RIN values (8.6, 8.4, 7.6, 5.9, 4.9 and 3.9) and performed RNA-Seq analysis. We measured the read coverage for all expressed genes as a function of transcript 3′ end distance (Fig. 2a, Additional file 2: Figure S2a) and observed that read coverage decreased as a function of both 3′ distance and sample RIN value. Furthermore, there was an exponential relationship between the median coverage and the distance from the 3′ end (Fig. 2a, Additional file 3: Table S2). The rate of exponential decay increased for highly degraded samples. We obtained similar results (Additional file 2: Figure S2b, Additional file 3: Table S2) in a replicate study using a public dataset [11] that had degraded and sequenced RNA from a U251 MG brain glioblastoma cell line using similar methods.

For a degraded sample we can increase the sequencing depth to obtain the coverage levels of an intact sample. We estimated this increase in sequencing depth as a function of the 3′ distance for the UHR sample at different levels of degradation (Additional file 2: Figure S2c). Given that the read coverage decreases exponentially as a function of the 3′ distance we have that even for a mildly degraded sample (RIN = 8.4) we need around 1.8x more reads to achieve adequate coverage at a distance of 2.5 kb from the 3′ end and around 4.9x more sequencing depth to achieve the same level of coverage at 5 kb from the 3′ end. For a highly degraded sample (RIN = 3.9) we need 9.4x and 28.3x more reads at a distance of 2.5 kb and 5 kb from the 3′ end, respectively. This fact makes the strategy of adding sequencing depth prohibitively expensive in practice.

### Coverage and decay profile of fusion related genes

We defined the median coverage decay rate per kilobase (decay rate for short) as the rate of decay of the median read coverage as a function of the distance from the 3′ end of the mRNA multiplied by 1 kb. The median read decay rates for the UHR and U251 samples at different RIN values are shown in Fig. 2b and similar median read decay rates were observed for independent samples with similar RIN values. This relationship can be modeled as *Median coverage decay rate*/*kb* =  −  1.45 + 0.13 *RIN* with an *R* ^2^ = 0.92. As expected, samples with lower RIN values had a larger negative decay rates and samples with high RIN values had a decay rate close to 0. It should be noted that there were samples that deviated from the model, for example our UHR sample with a RIN of 8.6 has a decay rate close to 0 even though it would have been expected to have a decay rate of approximately −0.2.

To characterize any bias associated with genes known to be part of cancer-related fusions, we queried all fusion partner genes in the COSMIC database [26]. Based on this gene set, the median distance of fusion breakpoints from the 3′ end is 2.7 kb, with approximately 80 % of the breakpoints occurring within 5 kb of the 3′ end and 95 % occurring within 7 kb of the 3′end (Fig. 2c). We then expanded this analysis to include fusion partners reported in the Atlas of Genetics and Cytogenetics in Oncology and Hematology [27] and scientific literature for potentially clinically significant gene fusions in solid and hematologic tumors as well as oncogenes not currently known to be involved in gene fusions that have the potential to be activated through gene fusions. This curated list of 545 genes (further referred to as 545 gene set) (Additional file 3: Table S3) has a median size of 3.8 kb (Additional file 4: Figure S3a), which is higher than the median size of 2.5 kb for all genes reported in UCSC (Additional file 4: Figure S3a). Evaluating the 545 gene set in the UHR sample data, we show the median coverage follows a similar exponential decay dependent on the distance from the 3′ end, but with a 12 % greater decay rate than that computed for all expressed genes (Additional file 4: Figure S3b).

To further validate our findings we isolated RNA from 20 samples, which included 14 tumor specimens (7 cell lines and 7 snap frozen tumor specimens) and 6 snap frozen normal tissue specimens (Additional file 3: Table S4). RNA from the 7 cell line samples had high RIN values (average = 9.9, minimum = 9.7, maximum = 10), while RNA from the 13 tumor and normal fresh tissue samples had lower RIN values (average 8.0, minimum = 3.4, maximum = 9.3). No intentional degradation was performed on these samples. We calculated the decay rate for the 545 gene set on these 20 samples (Additional file 3: Table S4, Fig. 2d). Reflective of our previous findings, there was a direct relationship between sample RIN and the decay rate modelled as *Median coverage decay rate*/*kb* = − 1.51 + 0.15 *RIN* with an. *R* ^2^ = 0.66. It should be noted that samples with similar RIN values did not always have the same decay rate and that individual samples deviated from the trend line. These differences were more pronounced for samples with lower RIN values. For example, the two most degraded samples (Additional file 3: Table S4) had very different RIN values (3.4 and 6.4) but similar decay rates (−0.74 and −0.76).

### The sensitivity of fusion detection depends on fusion breakpoint distance from the 3′ end

**Fig. 3:**
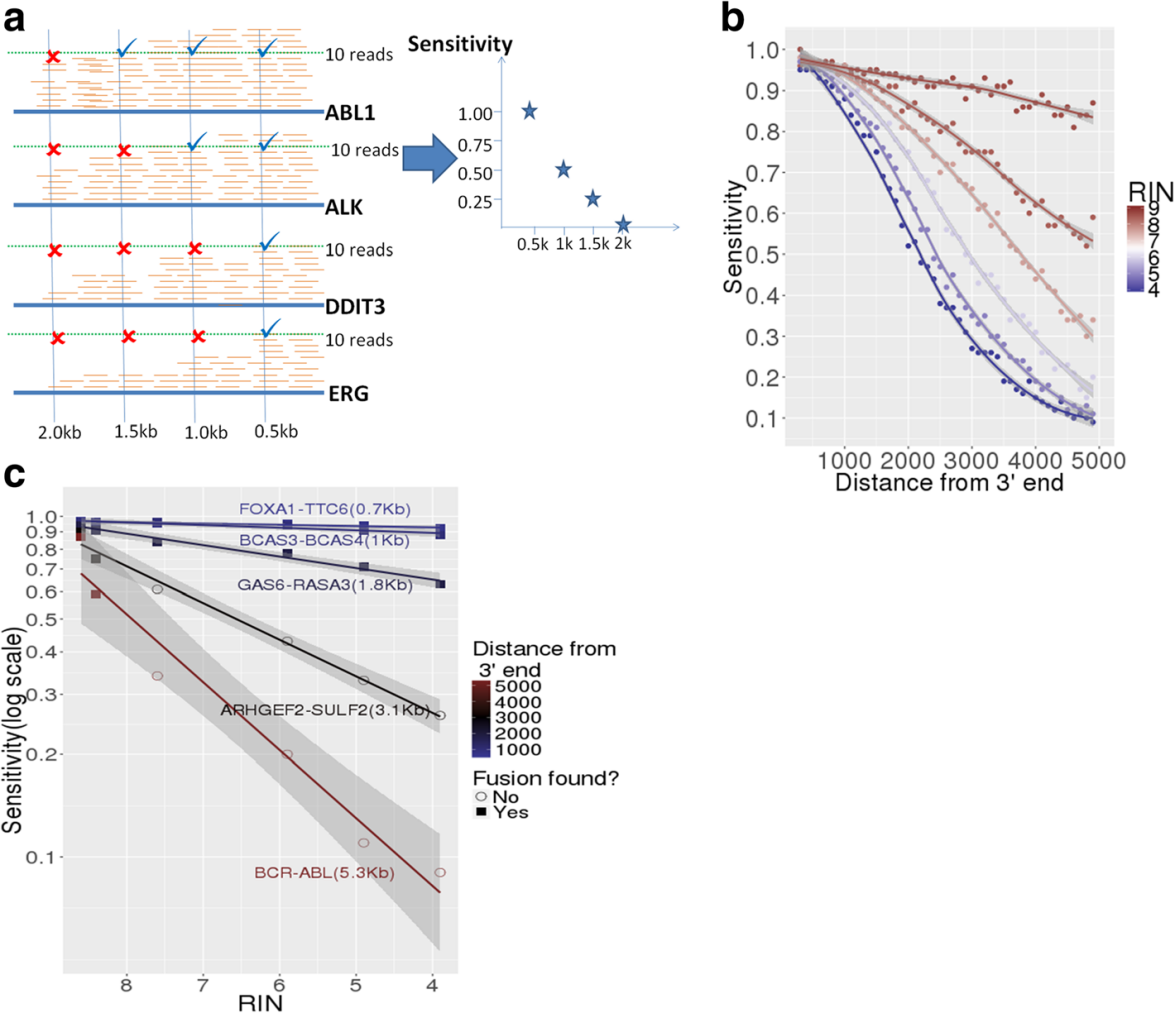
Estimation of fusion sensitivity as a function of distance of breakpoint from the 3′ end. **a** Diagram showing the strategy used to estimate the probability of detecting a fusion (i.e. sensitivity) at different distances from the 3′ end by enumerating the proportion of genes having a coverage ≥10x. A  indicates that there are more than 10 reads at that particular position for that gene while a X indicates that there are not. **b** Sensitivity as a function of the distance from the 3′ end for the UHR sample at varying levels of degradation. Loess trend is shown for each sample and 95 % confidence intervals are shown in *gray*. **c** Estimated sensitivity (log scale) for five different fusions present in UHR that occur at different distances from the 3′ end at different RIN values. If the fusion was detected at the particular degradation level it is shown as a *square* and if it is not detected it is shown as a *circle*. Linear trend line is shown for each fusion and 95 % confidence intervals are shown in *gray*

Using the 545 gene set, the probability of detecting a gene fusion (i.e. sensitivity on y-axis of plot) whose breakpoint occurs at a specific distance from the 3′ was determined by calculating the fraction of expressed genes whose coverage was ≥10x at that distance from the 3′ end of the gene (Fig. 3a). This calculation assumes at least 10 total reads are required to detect a heterozygous fusion product at our lower detection limit, which requires at least 5 supporting reads. Using the 545 gene set we plotted the estimated fusion detection sensitivity (Fig. 3b). The sensitivity decreased with the distance from the 3′ end, with greater reductions in sensitivity for the more heavily degraded UHR samples. However, the sensitivity remained high (>85 %) for all samples for fusion breakpoints less than 1 kb from the 3′ end regardless of RIN value.

The UHR is RNA isolated from 10 tumor cell lines [22], some which contain well characterized fusions (e.g. *BCR-ABL* fusion in the CML tumor cell line). We plotted the estimated sensitivity per RIN score for five known fusions in UHR with breakpoints having varied distances from the 3′ end of the gene (Fig. 3c); also computing whether that particular fusion had more than 5 supporting reads (Fig. 3c, Additional file 3: Table S5). As expected, the fusion detection for each of these 5 fusions is consistent with the estimated sensitivity, in that fusions with breakpoints further away from the 3′ end (e.g. *BCR-ABL* at 5.3 kb or *ARHGEF2-SULF2* at 3.1 kb) are not detected as the level of degradation increases. At the same time, fusions with breakpoint distance less than 1 kb to 3′end (e.g. *FOXA1-TTC6* or *BCAS3-BCAS4*) were detected regardless of RIN value (Fig. 3c). Interestingly, the number of supporting reads for these fusions increased as the RIN value decreased down to 5.9 (Additional file 3: Table S5), which is consistent with a majority of the reads coming from the 3′ end. No false positives were found at different levels of degradation.

We tested our approach on our previously described set of 20 specimens. Out of the 14 tumor samples from such set, 12 had known fusions. Using RNA-seq we were able to detect the known fusions from the 12 cases. We calculated the estimated sensitivity for the distance from the 3′ end of the breakpoint of each fusion in each sample (Additional file 3: Table S6). The fusions that were detected had high values of estimated sensitivity at its corresponding distance from the 3′ end (average = 92 %, minimum = 75 %, maximum = 96 %).

## Discussion

### RNA-Seq Poly-A pull down libraries impact fusion detection accuracy in degraded samples

In this study, we designed experiments using artificially degraded RNA from cell lines as well as naturally degraded RNA from tissue samples to quantify the effect RNA degradation has on fusion detection when using poly-A selected RNA libraries.

We found that both the RNA degradation level and the distance from the 3′ end of a gene, negatively impact the read coverage profile in RNA-seq. Furthermore, the median transcript coverage decreases exponentially as a function of the distance from the 3′ end and there is a linear relationship between the coverage decay rate and the RNA integrity number (RIN).

We identified a set of 545 genes that are putatively involved in fusions events. Using this gene set we calculated the probability of detecting a gene fusion (“sensitivity”) as a function of the distance of the fusion breakpoint from the 3′ end by calculating the fraction of expressed genes whose coverage was ≥10x at that distance from the 3′ end of the gene. The fusion detection sensitivity is negatively impacted by the sample degradation (measured by either the RIN value or the coverage decay rate) and the distance of the fusion breakpoint from the 3′ end of the fusion gene. Such effect, however, was not observed for fusions with breakpoints close to the 3′ end of genes, regardless of degradation level.

The fusion detection sensitivity can be affected by the choice of the aligner and the number of minimum supporting reads used to call a fusion event. In order to minimize the influence of a the combination of aligner and fusion detection tool parameters we provide a tool, Fusion Sense (http://bioinformaticstools.mayo.edu/research/fusion-sense), which given an alignment file in BAM format and a minimum coverage threshold, calculates the fusion detection sensitivity. Users are encouraged to use different aligners and coverage thresholds to mitigate the effect of the alignment tool and parameters used.

Methods that avoid the use of poly-A selection (e.g. RiboMinus™) do not suffer from the bias presented in this study [11, 20]. Nonetheless, the TruSeq methodology (which utilizes poly-A selected mRNA) is a commonly used method and large publically available RNA-seq reference datasets such as TCGA [9] and GTEX [10] have used this methodology. For example, the median RIN for samples from the TCGA for ovarian cancer was 8.4 [28], with close to 30 samples with a RIN below 7.0. Similarly a recent study [15] showed that brain samples from the GTEX study had a median RIN value of 7.2, including about 20 samples with a RIN below 6. Our study reveals that gene fusions with breakpoints distant from the 3′ end might be underrepresented in these datasets and extensive characterizations of these samples is left as future work.

### Using poly-A pull down libraries for fusion finding in the clinical setting

Clinical RNA-Seq assays that utilize poly-A selected RNA should provide some information in the report on the likelihood that particular gene fusions would be detected based on the level of degradation of the patients specimen. The degree of RNA degradation can be measured before library preparation with techniques such as the Agilent Bioanalyzer that provide RIN numbers. However, RNA quality can be better assessed after sequencing [14, 15]. In this paper we calculate this information by measuring the sample’s RNA decay rate.

For example, for a particular sample with a RIN of 6.0 and a decay rate similar to our UHR sample, the likelihood of detecting a gene fusion with a breakpoint at 1, 3, and 5 kb from the 3’end of the transcript would be 95, 45 and 20 % (Fig. 3b). So, if a clinician had sent such a sample from a patient suspected of having a *TMPRSS2-ERG* fusion (whose breakpoint occurs at around 5 kb from the 3′ end) and was interested in knowing if this gene fusion was present, the physician would understand the high risk of a false negative result (>50 %) given the degradation level of such specimen.

Our method can only be used to assess the sensitivity of fusion detection for a particular sample; however it does not increase the fusion sensitivity for such cases. It would be feasible to design fusion detection algorithms that decrease the read evidence needed as a function of the degradation and the distance of the breakpoint from the 3′ end and this is left as future work.

## Conclusions

In our present study, we found that when using poly-A pulldown techniques for library preparation in RNA-seq, the fusion detection sensitivity is negatively impacted by both sample degradation and distance of the fusion breakpoint from the 3′ end. We developed software that produces graphs that depict the effect on fusion sensitivity of sample degradation and 3′ end breakpoint distance. Such graphs can be useful in assessing the fusion detection sensitivity of RNA-seq in both research and clinical settings.

## Methods

### Samples

The 20 normal and tumor specimens involved in this study were collected and processed as part of the development and verification of a clinical test. All samples were de-identified and the publication of resultant data was approved by the Mayo Clinic Institutional Review Board. Total RNA was extracted from solid tumor tissue and whole blood using the Qiagen® miRNeasy Micro and Mini kits, respectively. Cell lines for this study were created from residual patient tumor tissue except Kasumi-1, KU812, (ATCC®) and Karpas 299 (Sigma-Aldrich®) which were obtained commercially. UHR (Universal Human Reference RNA) was purchased from Agilent (Santa Clara, CA). UHR is a mixture of cell lines derived from breast adenocarcinoma, hepatoblastoma, cervix adenocarcinoma, testis embryonal carcinoma, gliobastoma, melanoma, liposarcoma, histiocytic lymphoma, lymphoblastic leukemia and plasmocytoma. FASTQ sequencing files for Human U-251 MG brain glioblastoma cell lines (GBM) [11] were obtained from SRA under accession SRP023548.

### Controlled degradation experiments

Two micrograms of human universal reference RNA (UHR) (Agilent Technologies, Santa Clara, CA) and 1ug of RNA extracted from KU812 cell line (purchased from ATCC) were degraded at 74 °C for 1 to 11 min in 1 min intervals, using the NEBNext® Magnesium RNA Fragmentation Module Kit (NEB, Ipswich, MA). RNA was then purified and concentrated with RNeasy MinElute Cleanup Kit (Qiagen, Valencia, CA).

### Library preparation and next generation sequencing

RNA quality [RNA Integrity Number (RIN)] was assessed on an Agilent 2100 Bioanalyzer with the RNA 6000 Nano Kit and quantified with a Qubit® 2.0 fluorometer with the Qubit® RNA BR Assay Kit. The TruSeq® RNA Sample Preparation v2 Kit (Illumina, San Diego, CA) was used for isolation of polyadenylated mRNA with oligo-dT beads, second strand cDNA synthesis and NGS library preparation. Paired-end, 101 bp sequencing was performed on a HiSeq 2500 (Illumina) instrument in Rapid Run mode. Base calling was performed by the instrument computer using Illumina Real Time Analysis (RTA) software that is integrated with HiSeq Control Software (HCS) and provides a summary of quality statistics as per Illumina’s acceptance criteria for sequencing. CASAVA 1.8.2 was used for de-multiplexing and conversion of base calls to paired-end FASTQ files. Sequencing reads for the samples described are available from the Gene Expression Omnibus (GEO) under accession number GSE80126.

### Alignment and fusion detection methods

Individual coverage statistics for the samples analysed are available in Additional file 3: Table S1. RNA-seq data was analyzed using the MAP-RSeq [29] pipeline v 1.2 which is based on TopHat 2.0 [30] and a modified version of TopHat-Fusion 2.0.6 [4]. Reads were aligned to the human reference GRCh37 without alternative haplotypes. During alignment, TopHat was supplied with transcript models from UCSC (March 2012 version) available from Illumina’s iGenomes Project. Fusions reported required a minimum of 5 total read fusion events and to be within the exon-exon boundary of at least one of the genes from the 545 gene set. We also used STAR [16] 2.4.2a and STAR-fusion 0.6 and consider fusions with at least 5 supporting reads that were annotated as “ONLY_REF_SPLICE” and for which at least one of gene fusion partners was in the 545 gene set.

### Read coverage decay calculation

For the UHR samples read coverage was normalized to 50 million reads per sample. The utility DepthOfCoverage from the Genome Analysis Toolkit (GATK) v.1.6.7 was used to query the alignment files to obtain read depth coverage information. Coverage was normalized to coverage per million reads for each sample but refer to as “coverage” below. Only genes with coverage > 10x at a position 300 bases from the 3′ end were considered. The number of reads for each of these genes was measured starting at a position 300 bases from the 3′ end and every 100 bases after that. We denote by *c*(*x*) the median coverage for these genes at a distance *x* from the 3′ end. A linear model log(*c*(*x*)) = *mx* + *b* was built in R (version 3.1) and the decay rate per kilobase *d* was defined as *d* = *m* × 1000. Decay rate values and *R* ^2^ are available in Additional file 3: Table S2 for the UHR and U251 samples. A similar model was constructed by considering only genes from the 545 gene set. This was done for a set of 20 samples where decay rates and *R* ^2^ are reported in Additional file 3: Table S4. To model the effect of the decay rate and the RIN value we defined a linear model *d* = *m*′ × *RIN* + *b* ' in R (version 3.1) and the equations and the coefficients of the linear model and *R* ^2^ are reported in the results section.

## Additional files

Additional file 1: Figure S1. Read coverage profile for *BCR* and *ABL* in chemically degraded RNA from a KU812 cell line. A) Integrative Genomics Viewer (IGV) screenshot for the coverage profile across the *ABL1* gene for KU812 cell line at different levels of degradation. All samples were normalized to the same level of sequencing depth (13 million reads). The lower part of the figure shows an amplified view of exon 2 and exon 11. B) IGV screenshot for the coverage profile across the *BCR* gene for KU812 cell line at different levels of degradation. The lower part of the figure shows an amplified view of exon 15 and exon 23. (TIF 933 kb)
Additional file 2: Figure S2. Read coverage profile as a function of the distance from 3′ end for chemically degraded UHR and RNA isolated from cell lines. A) Box plot of the number of reads (in log scale) of a chemically degraded UHR sample at different RIN values (8.6, 8.4, 7.6, 5.9, 4.9 and 3.9) as a function of the distance from the 3′ end for all expressed genes. B) Median coverage per million reads (in log scale) as function of the distance from the 3′ end for RNA isolated from a U251 MG brain glioblastoma cell line at different RIN values (10,8,6,4). Individual linear trend lines are shown for each sample and 95 % confidence intervals are denoted in gray. Notice that there were replicates for each cell line at different RIN values. C) Increase in sequencing depth needed to achieve the coverage of an intact sample as a function of the distance from the 3′ end for the chemically degraded UHR sample at different RIN values (8.6, 8.4, 7.6, 5.9, 4.9 and 3.9). The increase in sequencing depth is calculated as the median coverage per million reads of an intact UHR (which is approximated by the median coverage per million reads of a UHR with a RIN = 8.6 at a distance of 300 bp from the 3′ end) divided by the median coverage per million reads of UHR at a particular RIN and distance from the 3′ end. (TIF 2280 kb)
Additional file 3: Table S1. Total read depth, percent of reads mapping to the human genome and average read depth on exome region for samples used. **Table S2.** RIN values, decay rates and ***R*** ^2^ and p values for the model log(*median coverage*) = *decay rate*/1000 × *distance* + *offset* for chemically degraded cell lines. UHR was chemically degraded in-house and U251 was available from the literature (9). **Table S3.** List of genes involved in fusions. Information for each gene includes gene symbol, transcript id and gene description. **Table S4.** RIN values, decay rates and ***R*** ^2^ and p values for the model log(*median coverage*) = *decay rate*/1000 × *distance* + *offset*, where median coverage is only calculated for expressed genes in the fusion list, for normal and tumor tissue samples with different levels of RNA degradation. Sample type (either tissue or cell line), histology and anatomical site is provided. **Table S5.** Number of fusion supporting reads per 50 million reads for different fusions occurring in UHR at different levels of RNA degradation. The distance of the breakpoint from the 3′ end is also provided. **Table S6.** List of fusions detected in tumor samples from Additional file 3 Table S3. The sample type, histologic diagnosis, RIN value, distance of the gene fusion breakpoint from the 3′ end, estimated sensitivity for the corresponding distance from the 3′ end to the breakpoint (calculated as in Fig. 3a) are also provided. For fusions whose distance is over 5 kb, we included the estimated sensitivity at 5 kb and denoted with the symbol *. (DOCX 69 kb)
Additional file 4: Figure S3. Statistics of genes involved in fusions. A) Cumulative distribution of the length for all genes and for genes involved in fusions. B) Plot of decay rates in UHR at different degradation values calculated across all genes (x-axis) and across only genes involved in fusions. Linear trend line is shown and 95 % confidence intervals are depicted in gray. The formula for the linear trend is *y* = 1.12*x* − 0.04. (TIF 535 kb)

## Abbreviations

CML: Chronic myelogenous leukemia
NGS: Next generation sequencing
RIN: RNA integrity number
UHR: Universal Human Reference RNA
