# Supplementary figures and images for: Impact of RNA degradation on fusion detection by RNA-seq
Source: BMC Genomics. 2016 Oct 20;17:814. doi: 10.1186/s12864-016-3161-9 (PMC5072325; doi:10.1186/s12864-016-3161-9)

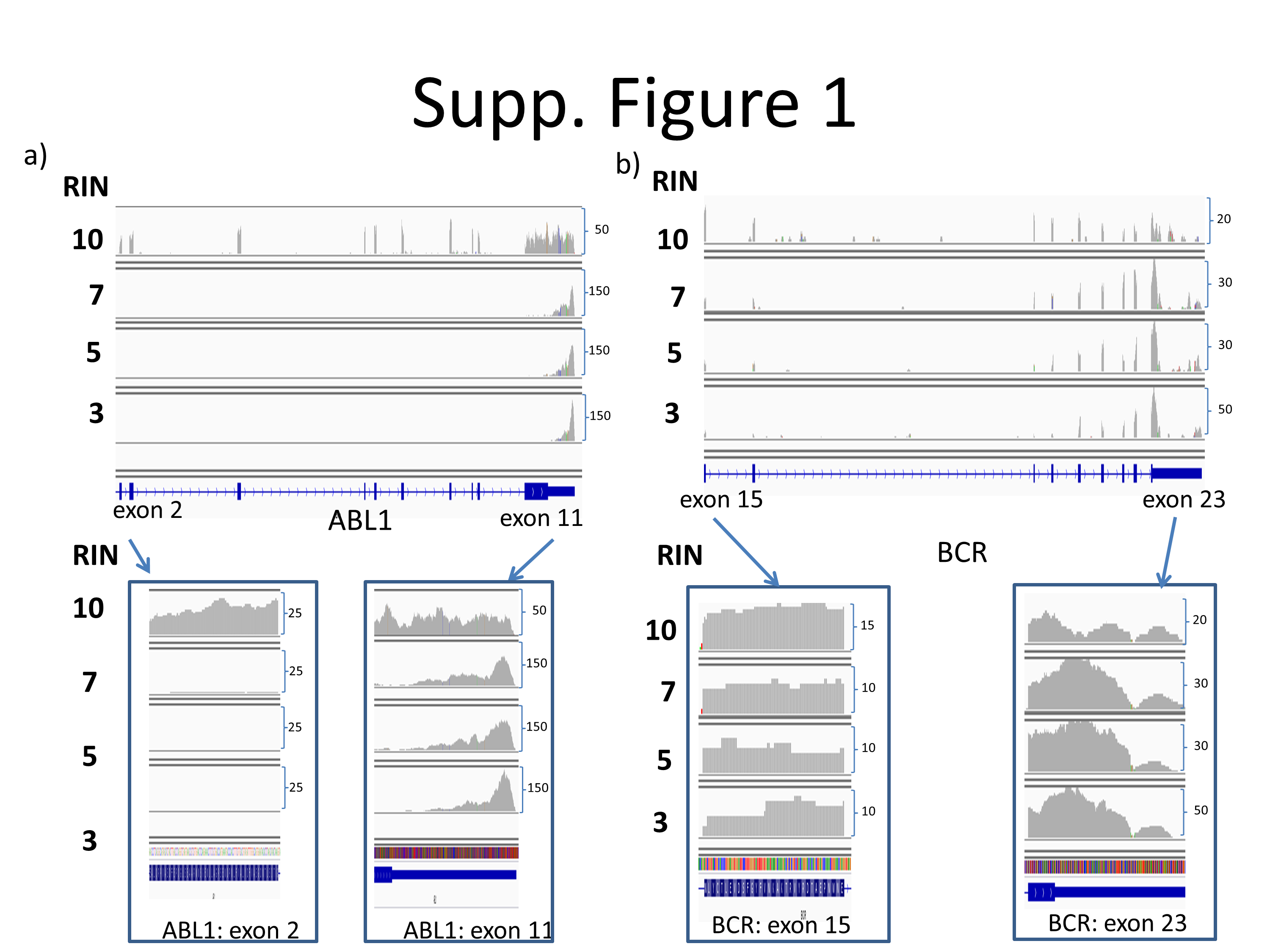

Supplement: Additional file 1: Figure S1. — Read coverage profile for BCR and ABL in chemically degraded RNA from a KU812 cell line. A) Integrative Genomics Viewer (IGV) screenshot for the coverage profile across the ABL1 gene for KU812 cell line at different levels of degradation. All samples were normalized to the same level of sequencing depth (13 million reads). The lower part of the figure shows an amplified view of exon 2 and exon 11. B) IGV screenshot for the coverage profile across the BCR gene for KU812 cell line at different levels of degradation. The lower part of the figure shows an amplified view of exon 15 and exon 23. (TIF 933 kb) [file 12864_2016_3161_MOESM1_ESM.tif]

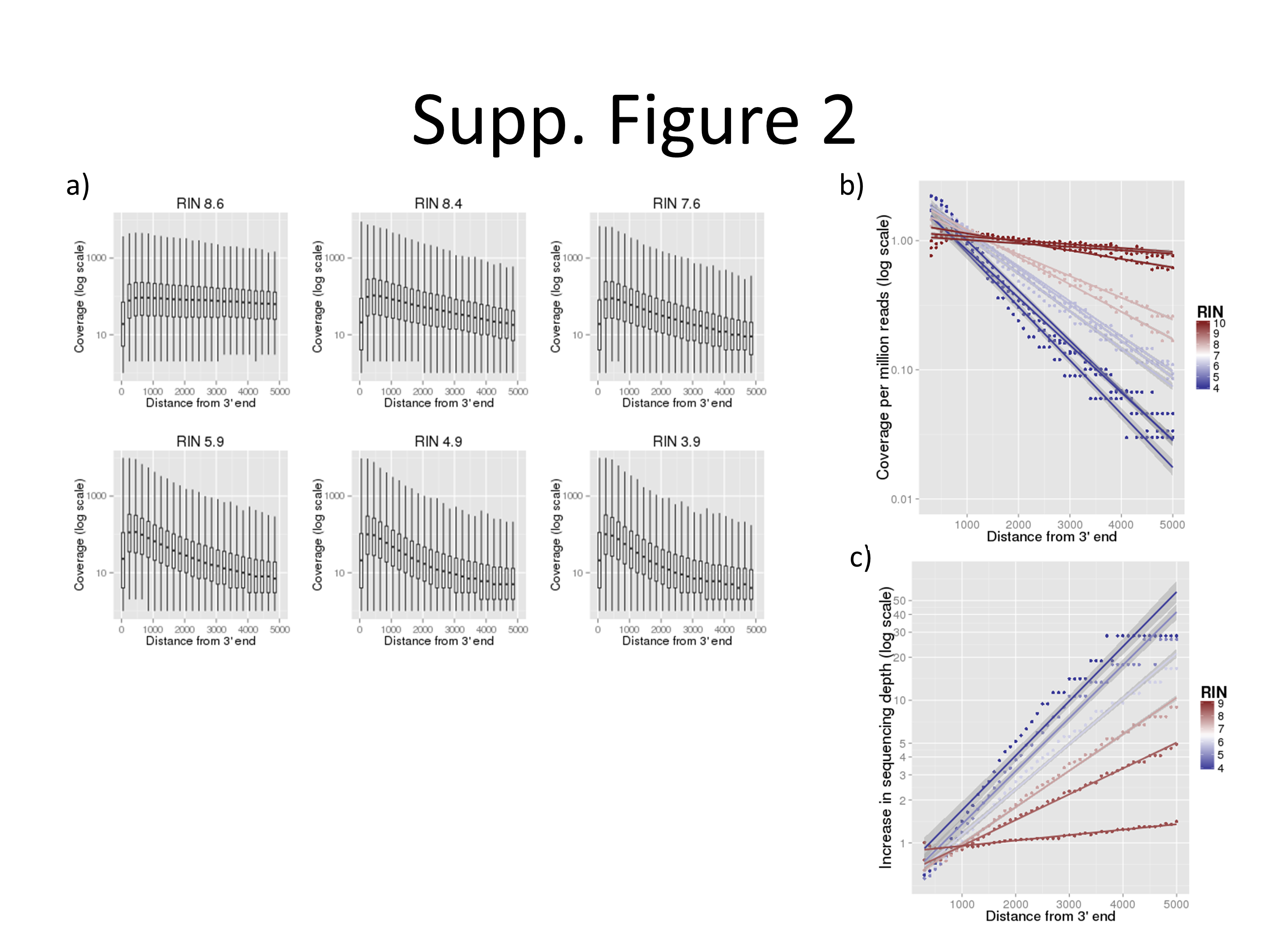

Supplement: Additional file 2: Figure S2. — Read coverage profile as a function of the distance from 3′ end for chemically degraded UHR and RNA isolated from cell lines. A) Box plot of the number of reads (in log scale) of a chemically degraded UHR sample at different RIN values (8.6, 8.4, 7.6, 5.9, 4.9 and 3.9) as a function of the distance from the 3′ end for all expressed genes. B) Median coverage per million reads (in log scale) as function of the distance from the 3′ end for RNA isolated from a U251 MG brain glioblastoma cell line at different RIN values (10,8,6,4). Individual linear trend lines are shown for each sample and 95 % confidence intervals are denoted in gray. Notice that there were replicates for each cell line at different RIN values. C) Increase in sequencing depth needed to achieve the coverage of an intact sample as a function of the distance from the 3′ end for the chemically degraded UHR sample at different RIN values (8.6, 8.4, 7.6, 5.9, 4.9 and 3.9). The increase in sequencing depth is calculated as the median coverage per million reads of an intact UHR (which is approximated by the median coverage per million reads of a UHR with a RIN = 8.6 at a distance of 300 bp from the 3′ end) divided by the median coverage per million reads of UHR at a particular RIN and distance from the 3′ end. (TIF 2280 kb) [file 12864_2016_3161_MOESM2_ESM.tif]

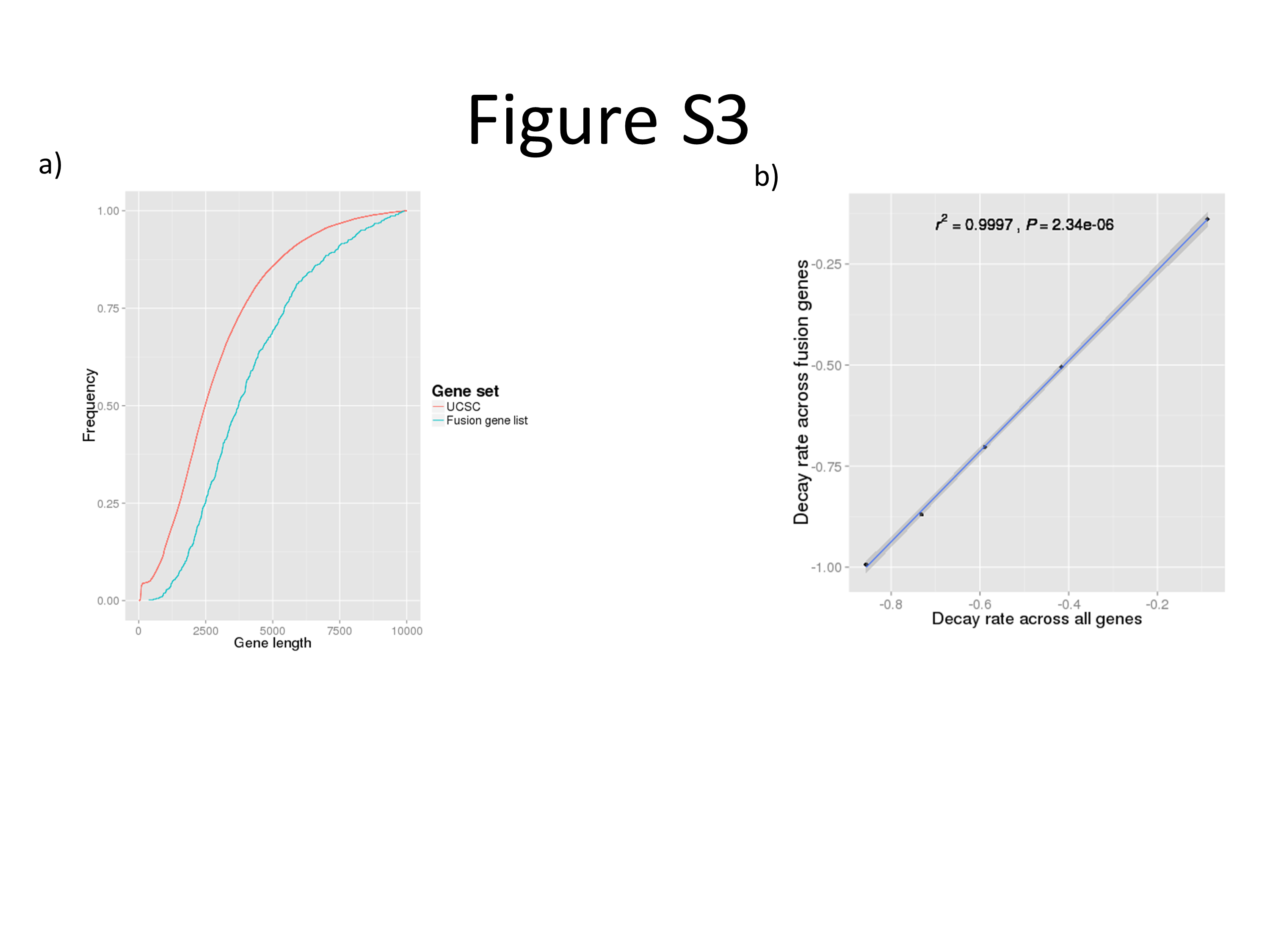

Supplement: Additional file 4: Figure S3. — Statistics of genes involved in fusions. A) Cumulative distribution of the length for all genes and for genes involved in fusions. B) Plot of decay rates in UHR at different degradation values calculated across all genes (x-axis) and across only genes involved in fusions. Linear trend line is shown and 95 % confidence intervals are depicted in gray. The formula for the linear trend is y = 1.12x − 0.04. (TIF 535 kb) [file 12864_2016_3161_MOESM4_ESM.tif]
