# Supplementary material for: Impact of RNA degradation on fusion detection by RNA-seq
Source: BMC Genomics. 2016 Oct 20;17:814. doi: 10.1186/s12864-016-3161-9 (PMC5072325; doi:10.1186/s12864-016-3161-9)
Supplement: Additional file 3: Table S1. — Total read depth, percent of reads mapping to the human genome and average read depth on exome region for samples used. Table S2. RIN values, decay rates and R 2 and p values for the model log(median coverage) = decay rate/1000 × distance + offset for chemically degraded cell lines. UHR was chemically degraded in-house and U251 was available from the literature (9). Table S3. List of genes involved in fusions. Information for each gene includes gene symbol, transcript id and gene description. Table S4. RIN values, decay rates and R 2 and p values for the model log(median coverage) = decay rate/1000 × distance + offset, where median coverage is only calculated for expressed genes in the fusion list, for normal and tumor tissue samples with different levels of RNA degradation. Sample type (either tissue or cell line), histology and anatomical site is provided. Table S5. Number of fusion supporting reads per 50 million reads for different fusions occurring in UHR at different levels of RNA degradation. The distance of the breakpoint from the 3′ end is also provided. Table S6. List of fusions detected in tumor samples from Additional file 3 Table S3. The sample type, histologic diagnosis, RIN value, distance of the gene fusion breakpoint from the 3′ end, estimated sensitivity for the corresponding distance from the 3′ end to the breakpoint (calculated as in Fig. 3a) are also provided. For fusions whose distance is over 5 kb, we included the estimated sensitivity at 5 kb and denoted with the symbol *. (DOCX 69 kb) [file 12864_2016_3161_MOESM3_ESM.docx]

**Supplementary Tables**

**Table S1.** Total read depth, percent of reads mapping to the human genome and average read depth on exome region for samples used.

| **Sample** | **Total read depth (Million reads)** | **Percent of reads mapping to hg19** | **Average read depth on exome region** |
| --- | --- | --- | --- |
| KU812 (RIN=10) | 124.62 | 94.9 | 291.48 |
| KU812 (RIN=7) | 13.45 | 93.0 | 30.84 |
| KU812 (RIN=5) | 17.75 | 93.4 | 40.86 |
| KU812 (RIN=3) | 16.16 | 93.5 | 37.20 |
| UHR (RIN=8.6) | 124.03 | 93.3 | 285.43 |
| UHR (RIN=8.4) | 113.42 | 93.3 | 261.05 |
| UHR (RIN=7.6) | 82.71 | 94.5 | 192.87 |
| UHR (RIN=5.9) | 102.95 | 93.5 | 237.42 |
| UHR (RIN=4.9) | 82.19 | 92.7 | 187.93 |
| UHR (RIN=3.9) | 87.08 | 92.0 | 197.47 |
| V1-12 | 90.96 | 94.8 | 212.62 |
| V1-24 | 133.29 | 95.8 | 314.92 |
| V1-25 | 93.28 | 96.0 | 220.89 |
| V1-30 | 111.05 | 92.9 | 254.28 |
| V1-31 | 122.48 | 95.9 | 289.66 |
| V1-34 | 101.09 | 95.7 | 238.60 |
| V1-35 | 83.87 | 94.2 | 194.90 |
| V1-36 | 91.69 | 94.7 | 214.03 |
| V1-4 | 154.74 | 93.6 | 357.11 |
| V1-6 | 123.24 | 95.5 | 290.19 |
| V3-41 | 120.36 | 94.1 | 279.42 |
| V3-43 | 139.39 | 96.4 | 331.33 |
| V3-44 | 133.26 | 94.5 | 310.40 |
| V3-45 | 144.04 | 94.5 | 335.81 |
| V3-59 | 73.45 | 95.4 | 172.73 |
| V3-60 | 97.62 | 95.2 | 229.09 |
| V3-61 | 72.50 | 69.06 | 170.30 |
| V3-62 | 116.33 | 93.1 | 267.18 |
| V3-63 | 102.7 | 94.7 | 239.94 |

**Table S2.** RIN values, decay rates and $\boldsymbol{R}^{\boldsymbol{2}}$ and p values for the model $\log\left( median coverage \right)=decay rate/1000\times distance+offset$for chemically degraded cell lines. UHR was chemically degraded in-house and U251 was available from the literature([9](#_ENREF_9)).

| **Sample** | **RIN** | **Decay rate** | $\boldsymbol{R}^{\boldsymbol{2}}$ | **p value** |
| --- | --- | --- | --- | --- |
| UHR | 8.6 | -0.09 | 0.96 | 8.51E-34 |
| UHR | 8.4 | -0.42 | 1.00 | 2.11E-57 |
| UHR | 7.6 | -0.59 | 0.99 | 3.66E-50 |
| UHR | 5.9 | -0.73 | 0.99 | 8.03E-44 |
| UHR | 4.9 | -0.86 | 0.97 | 3.86E-36 |
| UHR | 3.9 | -0.88 | 0.94 | 4.72E-30 |
| U251 | 10.0 | -0.16 | 0.97 | 2.06E-36 |
| U251 | 10.0 | -0.08 | 0.85 | 3.50E-20 |
| U251 | 10.0 | -0.07 | 0.79 | 7.38E-17 |
| U251 | 8.0 | -0.49 | 1.00 | 3.63E-55 |
| U251 | 8.0 | -0.39 | 0.99 | 4.86E-53 |
| U251 | 6.0 | -0.63 | 0.99 | 4.43E-51 |
| U251 | 6.0 | -0.67 | 0.98 | 7.33E-42 |
| U251 | 6.0 | -0.65 | 0.99 | 2.06E-49 |
| U251 | 4.0 | -0.89 | 0.98 | 1.08E-39 |
| U251 | 4.0 | -0.95 | 0.96 | 8.95E-34 |
| U251 | 4.0 | -0.84 | 0.98 | 3.82E-39 |

**Table S3.** List of genes involved in fusions. Information for each gene includes gene symbol, transcript id and gene description.

| **Gene Symbol** | **Transcript ID** | **Gene description** |
| --- | --- | --- |
| ABI1 | NM_005470 | abl-interactor 1 |
| ABL1 | NM_007313 | c-abl oncogene 1, non-receptor tyrosine kinase |
| ABL2 | NM_007314 | v-abl Abelson murine leukemia viral oncogene homolog 2 |
| ACBD6 | NM_032360 | acyl-CoA binding domain containing 6 |
| ACLY | NM_198830 | ATP citrate lyase |
| ACSL3 | NM_203372 | acyl-CoA synthetase long-chain family member 3 |
| ACSL6 | NM_015256 | acyl-CoA synthetase long-chain family member 6 |
| ACTB | NM_001101 | actin, beta |
| AFF1 | NM_005935 | AF4/FMR2 family, member 1 |
| AFF3 | NM_002285 | AF4/FMR2 family, member 3 |
| AFF4 | NM_014423 | AF4/FMR2 family, member 4 |
| AGK | NM_018238 | acylglycerol kinase |
| AGPAT5 | NM_018361 | 1-acylglycerol-3-phosphate O-acyltransferase 5 (lysophosphatidic acid acyltransferase, epsilon) |
| AGTRAP | NM_020350 | angiotensin II receptor-associated protein |
| AKAP13 | NM_144767 | A kinase (PRKA) anchor protein 13 |
| AKAP9 | NM_147185 | A kinase (PRKA) anchor protein (yotiao) 9 |
| AKT1 | NM_005163 | v-akt murine thymoma viral oncogene homolog 1 |
| AKT2 | NM_001626 | v-akt murine thymoma viral oncogene homolog 2 |
| ALDH2 | NM_001204889 | aldehyde dehydrogenase 2 family (mitochondrial) |
| ALK | NM_004304 | anaplastic lymphoma receptor tyrosine kinase |
| ANK3 | NM_020987 | ankyrin 3, node of Ranvier (ankyrin G) |
| AP3B1 | NM_003664 | adaptor-related protein complex 3, beta 1 subunit |
| AR | NM_001011645 | androgen receptor |
| ARAF | NM_001654 | v-raf murine sarcoma 3611 viral oncogene homolog |
| ARFIP1 | NM_014447 | ADP-ribosylation factor interacting protein 1 |
| ARHGAP26 | NM_015071 | Rho GTPase activating protein 26 |
| ARHGEF12 | NM_015313 | Rho guanine nucleotide exchange factor (GEF) 12 |
| ARNT | NM_178427 | aryl hydrocarbon receptor nuclear translocator |
| ASPSCR1 | NR_045351 | alveolar soft part sarcoma chromosome region, candidate 1 |
| ATF1 | NM_005171 | activating transcription factor 1 |
| ATG4C | NM_178221 | autophagy related 4C, cysteine peptidase |
| ATG5 | NM_004849 | autophagy related 5 |
| ATIC | NM_004044 | 5-aminoimidazole-4-carboxamide ribonucleotide formyltransferase/IMP cyclohydrolase |
| ATP8B2 | NM_020452 | ATPase, aminophospholipid transporter, class I, type 8B, member 2 |
| AXL | NM_021913 | AXL receptor tyrosine kinase |
| BAIAP2L1 | NM_018842 | BAI1-associated protein 2-like 1 |
| BBS9 | NM_198428 | Bardet-Biedl syndrome 9 |
| BCL10 | NM_003921 | B-cell CLL/lymphoma 10 |
| BCL11A | NM_138559 | B-cell CLL/lymphoma 11A (zinc finger protein) |
| BCL11B | NM_138576 | B-cell CLL/lymphoma 11B (zinc finger protein) |
| BCL2 | NM_000657 | B-cell CLL/lymphoma 2 |
| BCL3 | NM_005178 | B-cell CLL/lymphoma 3 |
| BCL6 | NM_001706 | B-cell CLL/lymphoma 6 |
| BCL7A | NM_020993 | B-cell CLL/lymphoma 7A |
| BCL9 | NM_004326 | B-cell CLL/lymphoma 9 |
| BCOR | NM_017745 | BCL6 corepressor |
| BCR | NM_021574 | breakpoint cluster region |
| BIRC3 | NM_182962 | baculoviral IAP repeat containing 3 |
| BRAF | NM_004333 | v-raf murine sarcoma viral oncogene homolog B1 |
| BRD3 | NM_007371 | bromodomain containing 3 |
| BRD4 | NM_058243 | bromodomain containing 4 |
| BTG1 | NM_001731 | B-cell translocation gene 1, anti-proliferative |
| BTK | NM_000061 | Bruton agammaglobulinemia tyrosine kinase |
| C15orf55 | NM_175741 | chromosome 15 open reading frame 55 |
| C2orf44 | NM_025203 | chromosome 2 open reading frame 44 |
| CAMTA1 | NR_038934 | calmodulin binding transcription activator 1 |
| CANT1 | NM_138793 | calcium activated nucleotidase 1 |
| CARD11 | NM_032415 | caspase recruitment domain family, member 11 |
| CARS | NR_036542 | cysteinyl-tRNA synthetase |
| CASC5 | NM_170589 | cancer susceptibility candidate 5 |
| CBFA2T3 | NM_175931 | core-binding factor, runt domain, alpha subunit 2; translocated to, 3 |
| CBFB | NM_022845 | core-binding factor, beta subunit |
| CBL | NM_005188 | Cbl proto-oncogene, E3 ubiquitin protein ligase |
| CBLB | NM_170662 | Cbl proto-oncogene, E3 ubiquitin protein ligase B |
| CBLC | NM_012116 | Cbl proto-oncogene, E3 ubiquitin protein ligase C |
| CCDC6 | NM_005436 | coiled-coil domain containing 6 |
| CCNB1IP1 | NM_182852 | cyclin B1 interacting protein 1, E3 ubiquitin protein ligase |
| CCND1 | NM_053056 | cyclin D1 |
| CCND2 | NM_001759 | cyclin D2 |
| CCND3 | NM_001760 | cyclin D3 |
| CCNE1 | NM_001238 | cyclin E1 |
| CCNY | NM_181698 | cyclin Y |
| CD274 | NM_014143 | CD274 molecule |
| CD74 | NM_004355 | CD74 molecule, major histocompatibility complex, class II invariant chain |
| CDH11 | NM_001797 | cadherin 11, type 2, OB-cadherin (osteoblast) |
| CDK4 | NM_000075 | cyclin-dependent kinase 4 |
| CDK6 | NM_001259 | cyclin-dependent kinase 6 |
| CDX1 | NM_001804 | caudal type homeobox 1 |
| CDX2 | NM_001265 | caudal type homeobox 2 |
| CHCHD7 | NM_024300 | coiled-coil-helix-coiled-coil-helix domain containing 7 |
| CHIC2 | NM_012110 | cysteine-rich hydrophobic domain 2 |
| CHN1 | NR_038133 | chimerin 1 |
| CHRM1 | NM_000738 | cholinergic receptor, muscarinic 1 |
| CIC | NM_015125 | capicua homolog (Drosophila) |
| CIITA | NM_000246 | class II, major histocompatibility complex, transactivator |
| CLP1 | NM_006831 | cleavage and polyadenylation factor I subunit 1 |
| CLRN3 | NM_152311 | clarin 3 |
| CLTC | NM_004859 | clathrin, heavy chain (Hc) |
| CNBP | NM_003418 | CCHC-type zinc finger, nucleic acid binding protein |
| CNTRL | NM_007018 | centriolin |
| COX6C | NM_004374 | cytochrome c oxidase subunit VIc |
| CREB1 | NM_134442 | cAMP responsive element binding protein 1 |
| CREB3L1 | NM_052854 | cAMP responsive element binding protein 3-like 1 |
| CREB3L2 | NM_194071 | cAMP responsive element binding protein 3-like 2 |
| CREBBP | NM_004380 | CREB binding protein |
| CRTC1 | NM_015321 | CREB regulated transcription coactivator 1 |
| CRTC3 | NM_022769 | CREB regulated transcription coactivator 3 |
| CSF1 | NM_172212 | colony stimulating factor 1 (macrophage) |
| CSF1R | NM_005211 | colony stimulating factor 1 receptor |
| CSF3R | NM_172313 | colony stimulating factor 3 receptor (granulocyte) |
| CTAGE5 | NM_203356 | CTAGE family, member 5 |
| CTNNB1 | NM_001904 | catenin (cadherin-associated protein), beta 1, 88kDa |
| CUTA | NM_015921 | cutA divalent cation tolerance homolog (E. coli) |
| CXCR4 | NM_003467 | chemokine (C-X-C motif) receptor 4 |
| CXCR7 | NM_020311 | chemokine (C-X-C motif) receptor 7 |
| CYP17A1 | NM_000102 | cytochrome P450, family 17, subfamily A, polypeptide 1 |
| CYP39A1 | NM_016593 | cytochrome P450, family 39, subfamily A, polypeptide 1 |
| DAZL | NM_001351 | deleted in azoospermia-like |
| DDB2 | NM_000107 | damage-specific DNA binding protein 2, 48kDa |
| DDIT3 | NM_004083 | DNA-damage-inducible transcript 3 |
| DDX10 | NM_004398 | DEAD (Asp-Glu-Ala-Asp) box polypeptide 10 |
| DDX5 | NM_004396 | DEAD (Asp-Glu-Ala-Asp) box helicase 5 |
| DDX6 | NM_004397 | DEAD (Asp-Glu-Ala-Asp) box helicase 6 |
| DEK | NM_003472 | DEK oncogene |
| DHFR | NM_000791 | dihydrofolate reductase |
| DNAJB1 | NM_006145 | DnaJ (Hsp40) homolog, subfamily B, member 1 |
| DUSP22 | NM_020185 | dual specificity phosphatase 22 |
| EBF1 | NM_024007 | early B-cell factor 1 |
| EGFR | NM_201284 | epidermal growth factor receptor |
| EHF | NM_012153 | ets homologous factor |
| EIF3E | NM_001568 | eukaryotic translation initiation factor 3, subunit E |
| EIF3K | NM_013234 | eukaryotic translation initiation factor 3, subunit K |
| ELF4 | NM_001421 | E74-like factor 4 (ets domain transcription factor) |
| ELK4 | NM_021795 | ELK4, ETS-domain protein (SRF accessory protein 1) |
| ELL | NM_006532 | elongation factor RNA polymerase II |
| ELN | NM_001081755 | elastin |
| EML4 | NM_019063 | echinoderm microtubule associated protein like 4 |
| EP300 | NM_001429 | E1A binding protein p300 |
| EPC1 | NM_025209 | enhancer of polycomb homolog 1 (Drosophila) |
| EPHA2 | NM_004431 | EPH receptor A2 |
| EPOR | NR_033663 | erythropoietin receptor |
| EPS15 | NM_001981 | epidermal growth factor receptor pathway substrate 15 |
| ERBB2 | NM_004448 | v-erb-b2 erythroblastic leukemia viral oncogene homolog 2, neuro/glioblastoma derived oncogene homolog (avian) |
| ERBB3 | NM_001982 | v-erb-b2 erythroblastic leukemia viral oncogene homolog 3 (avian) |
| ERBB4 | NM_005235 | v-erb-a erythroblastic leukemia viral oncogene homolog 4 (avian) |
| ERC1 | NR_027949 | ELKS/RAB6-interacting/CAST family member 1 |
| ERG | NM_182918 | v-ets erythroblastosis virus E26 oncogene homolog (avian) |
| ERO1L | NM_014584 | ERO1-like (S. cerevisiae) |
| ESR1 | NM_001122742 | estrogen receptor 1 |
| ESRP1 | NM_017697 | epithelial splicing regulatory protein 1 |
| ETS1 | NM_005238 | v-ets erythroblastosis virus E26 oncogene homolog 1 (avian) |
| ETV1 | NM_004956 | ets variant 1 |
| ETV4 | NM_001986 | ets variant 4 |
| ETV5 | NM_004454 | ets variant 5 |
| ETV6 | NM_001987 | ets variant 6 |
| EWSR1 | NM_013986 | Ewing sarcoma breakpoint region 1 |
| EZH2 | NM_152998 | enhancer of zeste homolog 2 (Drosophila) |
| FAM114A2 | NM_018691 | family with sequence similarity 114, member A2 |
| FAM131B | NM_014690 | family with sequence similarity 131, member B |
| FAM22A | NM_001099338 | family with sequence similarity 22, member A |
| FAM86C1 | NM_152563 | family with sequence similarity 86, member C1 |
| FBXL18 | NM_024963 | F-box and leucine-rich repeat protein 18 |
| FBXO38 | NM_205836 | F-box protein 38 |
| FCHSD1 | NM_033449 | FCH and double SH3 domains 1 |
| FCRL4 | NM_031282 | Fc receptor-like 4 |
| FERMT2 | NM_006832 | fermitin family member 2 |
| FEV | NM_017521 | FEV (ETS oncogene family) |
| FGFR1 | NM_023110 | fibroblast growth factor receptor 1 |
| FGFR1OP | NM_194429 | FGFR1 oncogene partner |
| FGFR2 | NM_022970 | fibroblast growth factor receptor 2 |
| FGFR3 | NM_022965 | fibroblast growth factor receptor 3 |
| FGFR4 | NM_213647 | fibroblast growth factor receptor 4 |
| FGR | NM_005248 | Gardner-Rasheed feline sarcoma viral (v-fgr) oncogene homolog |
| FHDC1 | NM_033393 | FH2 domain containing 1 |
| FHIT | NM_002012 | fragile histidine triad |
| FIP1L1 | NM_030917 | FIP1 like 1 (S. cerevisiae) |
| FKBP15 | NM_015258 | FK506 binding protein 15, 133kDa |
| FKBP1A | NM_054014 | FK506 binding protein 1A, 12kDa |
| FLI1 | NM_002017 | Friend leukemia virus integration 1 |
| FLJ27352 | NM_001198784 | . |
| FLT1 | NM_002019 | fms-related tyrosine kinase 1 (vascular endothelial growth factor/vascular permeability factor receptor) |
| FLT3 | NM_004119 | fms-related tyrosine kinase 3 |
| FLT4 | NM_182925 | fms-related tyrosine kinase 4 |
| FN1 | NM_212482 | fibronectin 1 |
| FNBP1 | NM_015033 | formin binding protein 1 |
| FOXA1 | NM_004496 | forkhead box A1 |
| FOXO1 | NM_002015 | forkhead box O1 |
| FOXO3 | NM_201559 | forkhead box O3 |
| FOXO4 | NM_005938 | forkhead box O4 |
| FOXP1 | NM_032682 | forkhead box P1 |
| FSTL3 | NM_005860 | follistatin-like 3 (secreted glycoprotein) |
| FUS | NR_028388 | fused in sarcoma |
| FYN | NM_153048 | FYN oncogene related to SRC, FGR, YES |
| GAB2 | NM_080491 | GRB2-associated binding protein 2 |
| GABBR2 | NM_005458 | gamma-aminobutyric acid (GABA) B receptor, 2 |
| GAS7 | NM_201433 | growth arrest-specific 7 |
| GEMIN2 | NM_003616 | gem (nuclear organelle) associated protein 2 |
| GLI1 | NM_005269 | GLI family zinc finger 1 |
| GMDS | NM_001500 | GDP-mannose 4,6-dehydratase |
| GMPS | NM_003875 | guanine monphosphate synthetase |
| GNA11 | NM_002067 | guanine nucleotide binding protein (G protein), alpha 11 (Gq class) |
| GNAI1 | NM_002069 | guanine nucleotide binding protein (G protein), alpha inhibiting activity polypeptide 1 |
| GNAS | NR_003259 | GNAS complex locus |
| GNRHR | NM_001012763 | gonadotropin-releasing hormone receptor |
| GOLGA5 | NM_005113 | golgin A5 |
| GOPC | NM_020399 | golgi-associated PDZ and coiled-coil motif containing |
| GPBP1L1 | NM_021639 | GC-rich promoter binding protein 1-like 1 |
| GPHN | NM_020806 | gephyrin |
| GRHL2 | NM_024915 | grainyhead-like 2 (Drosophila) |
| GTF2IRD1 | NM_016328 | GTF2I repeat domain containing 1 |
| HAS2 | NM_005328 | hyaluronan synthase 2 |
| HCK | NM_002110 | hemopoietic cell kinase |
| HERPUD1 | NM_014685 | homocysteine-inducible, endoplasmic reticulum stress-inducible, ubiquitin-like domain member 1 |
| HEY1 | NM_012258 | hairy/enhancer-of-split related with YRPW motif 1 |
| HIP1 | NM_005338 | huntingtin interacting protein 1 |
| HIST1H4I | NM_003495 | histone cluster 1, H4i |
| HLF | NM_002126 | hepatic leukemia factor |
| HMGA1 | NM_145905 | high mobility group AT-hook 1 |
| HMGA2 | NM_003484 | high mobility group AT-hook 2 |
| HMGN2P46 | NR_022014 | high mobility group nucleosomal binding domain 2 pseudogene 46 |
| HN1 | NM_016185 | hematological and neurological expressed 1 |
| HOOK3 | NM_032410 | hook homolog 3 (Drosophila) |
| HOXA11 | NM_005523 | homeobox A11 |
| HOXA13 | NM_000522 | homeobox A13 |
| HOXA3 | NM_153631 | homeobox A3 |
| HOXA9 | NM_152739 | homeobox A9 |
| HOXC11 | NM_014212 | homeobox C11 |
| HOXC13 | NM_017410 | homeobox C13 |
| HOXD11 | NM_021192 | homeobox D11 |
| HOXD13 | NM_000523 | homeobox D13 |
| HRAS | NM_176795 | v-Ha-ras Harvey rat sarcoma viral oncogene homolog |
| IDH1 | NM_005896 | isocitrate dehydrogenase 1 (NADP+), soluble |
| IDH2 | NM_002168 | isocitrate dehydrogenase 2 (NADP+), mitochondrial |
| IGF2BP3 | NM_006547 | insulin-like growth factor 2 mRNA binding protein 3 |
| IKZF1 | NM_006060 | IKAROS family zinc finger 1 (Ikaros) |
| IL2 | NM_000586 | interleukin 2 |
| IL21R | NM_181079 | interleukin 21 receptor |
| IL3 | NM_000588 | interleukin 3 (colony-stimulating factor, multiple) |
| IL6R | NM_181359 | interleukin 6 receptor |
| IL7R | NM_002185 | interleukin 7 receptor |
| INTS4 | NM_033547 | integrator complex subunit 4 |
| IRF2BP2 | NM_182972 | interferon regulatory factor 2 binding protein 2 |
| IRF4 | NR_046000 | interferon regulatory factor 4 |
| ITK | NM_005546 | IL2-inducible T-cell kinase |
| ITPR2 | NM_002223 | inositol 1,4,5-trisphosphate receptor, type 2 |
| JAK1 | NM_002227 | Janus kinase 1 |
| JAK2 | NM_004972 | Janus kinase 2 |
| JAK3 | NM_000215 | Janus kinase 3 |
| JAZF1 | NM_175061 | JAZF zinc finger 1 |
| JUN | NM_002228 | jun proto-oncogene |
| KAT6A | NM_006766 | K(lysine) acetyltransferase 6A |
| KAT6B | NM_012330 | K(lysine) acetyltransferase 6B |
| KDM5A | NM_001042603 | lysine (K)-specific demethylase 5A |
| KDR | NM_002253 | kinase insert domain receptor (a type III receptor tyrosine kinase) |
| KDSR | NM_002035 | 3-ketodihydrosphingosine reductase |
| KIAA1549 | NM_020910 | KIAA1549 |
| KIF5B | NM_004521 | kinesin family member 5B |
| KIT | NM_001093772 | v-kit Hardy-Zuckerman 4 feline sarcoma viral oncogene homolog |
| KITLG | NM_003994 | KIT ligand |
| KLC1 | NM_182923 | kinesin light chain 1 |
| KLK2 | NR_045763 | kallikrein-related peptidase 2 |
| KRAS | NM_033360 | v-Ki-ras2 Kirsten rat sarcoma viral oncogene homolog |
| KTN1 | NM_182926 | kinectin 1 (kinesin receptor) |
| LASP1 | NM_006148 | LIM and SH3 protein 1 |
| LCK | NM_005356 | lymphocyte-specific protein tyrosine kinase |
| LCP1 | NM_002298 | lymphocyte cytosolic protein 1 (L-plastin) |
| LGR5 | NM_003667 | leucine-rich repeat containing G protein-coupled receptor 5 |
| LHFP | NM_005780 | lipoma HMGIC fusion partner |
| LIFR | NM_002310 | leukemia inhibitory factor receptor alpha |
| LMO1 | NM_002315 | LIM domain only 1 (rhombotin 1) |
| LMO2 | NM_005574 | LIM domain only 2 (rhombotin-like 1) |
| LPP | NM_005578 | LIM domain containing preferred translocation partner in lipoma |
| LRIG3 | NM_153377 | leucine-rich repeats and immunoglobulin-like domains 3 |
| LTK | NM_206961 | leukocyte receptor tyrosine kinase |
| LYL1 | NM_005583 | lymphoblastic leukemia derived sequence 1 |
| LYN | NM_002350 | v-yes-1 Yamaguchi sarcoma viral related oncogene homolog |
| MACF1 | NM_012090 | microtubule-actin crosslinking factor 1 |
| MAF | NM_005360 | v-maf musculoaponeurotic fibrosarcoma oncogene homolog (avian) |
| MAFB | NM_005461 | v-maf musculoaponeurotic fibrosarcoma oncogene homolog B (avian) |
| MALT1 | NM_173844 | mucosa associated lymphoid tissue lymphoma translocation gene 1 |
| MAML2 | NM_032427 | mastermind-like 2 (Drosophila) |
| MAML3 | NM_018717 | mastermind-like 3 (Drosophila) |
| MAP2K1 | NM_002755 | mitogen-activated protein kinase kinase 1 |
| MAP2K2 | NM_030662 | mitogen-activated protein kinase kinase 2 |
| MAP4K5 | NM_198794 | mitogen-activated protein kinase kinase kinase kinase 5 |
| MAST1 | NM_014975 | microtubule associated serine/threonine kinase 1 |
| MAST2 | NM_015112 | microtubule associated serine/threonine kinase 2 |
| MBOAT2 | NM_138799 | membrane bound O-acyltransferase domain containing 2 |
| MBTD1 | NM_017643 | mbt domain containing 1 |
| MCL1 | NM_182763 | myeloid cell leukemia sequence 1 (BCL2-related) |
| MCPH1 | NM_024596 | microcephalin 1 |
| MDM2 | NM_002392 | Mdm2, p53 E3 ubiquitin protein ligase homolog (mouse) |
| MDS2 | NR_027042 | myelodysplastic syndrome 2 translocation associated |
| MEAF6 | NM_022756 | MYST/Esa1-associated factor 6 |
| MECOM | NM_005241 | MDS1 and EVI1 complex locus |
| MED12 | NM_005120 | mediator complex subunit 12 |
| MET | NM_001127500 | met proto-oncogene (hepatocyte growth factor receptor) |
| MITF | NM_198178 | microphthalmia-associated transcription factor |
| MKL1 | NM_020831 | megakaryoblastic leukemia (translocation) 1 |
| MKRN1 | NM_013446 | makorin ring finger protein 1 |
| MLF1 | NM_022443 | myeloid leukemia factor 1 |
| MLLT1 | NM_005934 | myeloid/lymphoid or mixed-lineage leukemia (trithorax homolog, Drosophila); translocated to, 1 |
| MLLT10 | NM_004641 | myeloid/lymphoid or mixed-lineage leukemia (trithorax homolog, Drosophila); translocated to, 10 |
| MLLT11 | NM_006818 | myeloid/lymphoid or mixed-lineage leukemia (trithorax homolog, Drosophila); translocated to, 11 |
| MLLT3 | NM_004529 | myeloid/lymphoid or mixed-lineage leukemia (trithorax homolog, Drosophila); translocated to, 3 |
| MLLT4 | NM_001207008 | myeloid/lymphoid or mixed-lineage leukemia (trithorax homolog, Drosophila); translocated to, 4 |
| MLLT6 | NM_005937 | myeloid/lymphoid or mixed-lineage leukemia (trithorax homolog, Drosophila); translocated to, 6 |
| MN1 | NM_002430 | meningioma (disrupted in balanced translocation) 1 |
| MNX1 | NM_005515 | motor neuron and pancreas homeobox 1 |
| MPL | NM_005373 | myeloproliferative leukemia virus oncogene |
| MSI2 | NM_170721 | musashi homolog 2 (Drosophila) |
| MST1R | NM_002447 | macrophage stimulating 1 receptor (c-met-related tyrosine kinase) |
| MTCP1 | NM_001018025 | mature T-cell proliferation 1 |
| MTOR | NM_004958 | mechanistic target of rapamycin (serine/threonine kinase) |
| MUC1 | NM_002456 | mucin 1, cell surface associated |
| MYB | NM_005375 | v-myb myeloblastosis viral oncogene homolog (avian) |
| MYC | NM_002467 | v-myc myelocytomatosis viral oncogene homolog (avian) |
| MYCL1 | NM_005376 | v-myc myelocytomatosis viral oncogene homolog 1, lung carcinoma derived (avian) |
| MYCN | NM_005378 | v-myc myelocytomatosis viral related oncogene, neuroblastoma derived (avian) |
| MYD88 | NM_002468 | myeloid differentiation primary response gene (88) |
| MYH11 | NM_022844 | myosin, heavy chain 11, smooth muscle |
| NAB2 | NM_005967 | NGFI-A binding protein 2 (EGR1 binding protein 2) |
| NACC2 | NM_144653 | NACC family member 2, BEN and BTB (POZ) domain containing |
| NBEAP1 | NR_027992 | neurobeachin pseudogene 1 |
| NCKIPSD | NM_184231 | NCK interacting protein with SH3 domain |
| NCOA1 | NM_147233 | nuclear receptor coactivator 1 |
| NCOA2 | NM_006540 | nuclear receptor coactivator 2 |
| NDRG1 | NM_006096 | N-myc downstream regulated 1 |
| NFATC1 | NM_172390 | nuclear factor of activated T-cells, cytoplasmic, calcineurin-dependent 1 |
| NFATC2 | NM_173091 | nuclear factor of activated T-cells, cytoplasmic, calcineurin-dependent 2 |
| NFIA | NM_005595 | nuclear factor I/A |
| NFIB | NM_005596 | nuclear factor I/B |
| NFIX | NM_002501 | nuclear factor I/X (CCAAT-binding transcription factor) |
| NFKB2 | NM_002502 | nuclear factor of kappa light polypeptide gene enhancer in B-cells 2 (p49/p100) |
| NIN | NM_182946 | ninein (GSK3B interacting protein) |
| NKAIN2 | NM_153355 | Na+/K+ transporting ATPase interacting 2 |
| NKX2-1 | NM_003317 | NK2 homeobox 1 |
| NOTCH1 | NM_017617 | notch 1 |
| NOTCH2 | NM_024408 | notch 2 |
| NPM1 | NM_199185 | nucleophosmin (nucleolar phosphoprotein B23, numatrin) |
| NR4A3 | NM_173200 | nuclear receptor subfamily 4, group A, member 3 |
| NRAS | NM_002524 | neuroblastoma RAS viral (v-ras) oncogene homolog |
| NSD1 | NM_172349 | nuclear receptor binding SET domain protein 1 |
| NT5C2 | NM_012229 | 5'-nucleotidase, cytosolic II |
| NTN1 | NM_004822 | netrin 1 |
| NTRK1 | NM_002529 | neurotrophic tyrosine kinase, receptor, type 1 |
| NTRK2 | NM_006180 | neurotrophic tyrosine kinase, receptor, type 2 |
| NTRK3 | NM_002530 | neurotrophic tyrosine kinase, receptor, type 3 |
| NUMA1 | NM_006185 | nuclear mitotic apparatus protein 1 |
| NUP107 | NM_020401 | nucleoporin 107kDa |
| NUP214 | NM_005085 | nucleoporin 214kDa |
| NUP98 | NM_139132 | nucleoporin 98kDa |
| OFD1 | NM_003611 | oral-facial-digital syndrome 1 |
| OLIG2 | NM_005806 | oligodendrocyte lineage transcription factor 2 |
| OMD | NM_005014 | osteomodulin |
| PACS1 | NM_018026 | phosphofurin acidic cluster sorting protein 1 |
| PAFAH1B2 | NM_002572 | platelet-activating factor acetylhydrolase 1b, catalytic subunit 2 (30kDa) |
| PARP1 | NM_001618 | poly (ADP-ribose) polymerase 1 |
| PARP2 | NM_005484 | poly (ADP-ribose) polymerase 2 |
| PARP3 | NM_005485 | poly (ADP-ribose) polymerase family, member 3 |
| PATZ1 | NM_032052 | POZ (BTB) and AT hook containing zinc finger 1 |
| PAX3 | NM_181461 | paired box 3 |
| PAX5 | NM_016734 | paired box 5 |
| PAX7 | NM_013945 | paired box 7 |
| PBX1 | NM_002585 | pre-B-cell leukemia homeobox 1 |
| PCM1 | NM_006197 | pericentriolar material 1 |
| PCSK7 | NM_004716 | proprotein convertase subtilisin/kexin type 7 |
| PDCD1LG2 | NM_025239 | programmed cell death 1 ligand 2 |
| PDE8B | NM_003719 | phosphodiesterase 8B |
| PDGFA | NM_033023 | platelet-derived growth factor alpha polypeptide |
| PDGFB | NM_033016 | platelet-derived growth factor beta polypeptide |
| PDGFRA | NM_006206 | platelet-derived growth factor receptor, alpha polypeptide |
| PDGFRB | NM_002609 | platelet-derived growth factor receptor, beta polypeptide |
| PER1 | NM_002616 | period homolog 1 (Drosophila) |
| PHF1 | NR_027692 | PHD finger protein 1 |
| PICALM | NM_007166 | phosphatidylinositol binding clathrin assembly protein |
| PIK3CA | NM_006218 | phosphatidylinositol-4,5-bisphosphate 3-kinase, catalytic subunit alpha |
| PIK3CD | NM_005026 | phosphatidylinositol-4,5-bisphosphate 3-kinase, catalytic subunit delta |
| PIK3R1 | NM_181524 | phosphoinositide-3-kinase, regulatory subunit 1 (alpha) |
| PIM1 | NM_002648 | pim-1 oncogene |
| PKD1L1 | NM_138295 | polycystic kidney disease 1 like 1 |
| PLA2R1 | NM_007366 | phospholipase A2 receptor 1, 180kDa |
| PLAG1 | NM_002655 | pleiomorphic adenoma gene 1 |
| PLXND1 | NM_015103 | plexin D1 |
| PML | NM_033250 | promyelocytic leukemia |
| POR | NM_000941 | P450 (cytochrome) oxidoreductase |
| POU2AF1 | NM_006235 | POU class 2 associating factor 1 |
| POU5F1 | NM_203289 | POU class 5 homeobox 1 |
| PPARG | NM_138712 | peroxisome proliferator-activated receptor gamma |
| PPFIBP1 | NM_177444 | PTPRF interacting protein, binding protein 1 (liprin beta 1) |
| PPP1CB | NM_206876 | protein phosphatase 1, catalytic subunit, beta isozyme |
| PRCC | NM_005973 | papillary renal cell carcinoma (translocation-associated) |
| PRDM16 | NM_199454 | PR domain containing 16 |
| PRKACA | NM_207518 | protein kinase, cAMP-dependent, catalytic, alpha |
| PRKAR2A | NM_004157 | protein kinase, cAMP-dependent, regulatory, type II, alpha |
| PRKCE | NM_005400 | protein kinase C, epsilon |
| PRRX1 | NM_022716 | paired related homeobox 1 |
| PSIP1 | NM_033222 | PC4 and SFRS1 interacting protein 1 |
| PTBP3 | NM_005156 | polypyrimidine tract binding protein 3 |
| PTCH1 | NM_001083607 | patched 1 |
| PTK6 | NM_005975 | PTK6 protein tyrosine kinase 6 |
| PTK7 | NM_152882 | PTK7 protein tyrosine kinase 7 |
| PTPN11 | NM_002834 | protein tyrosine phosphatase, non-receptor type 11 |
| PTPRK | NM_002844 | protein tyrosine phosphatase, receptor type, K |
| QKI | NM_206855 | QKI, KH domain containing, RNA binding |
| RABEP1 | NM_004703 | rabaptin, RAB GTPase binding effector protein 1 |
| RAD51B | NM_133510 | RAD51 homolog B (S. cerevisiae) |
| RAF1 | NM_002880 | v-raf-1 murine leukemia viral oncogene homolog 1 |
| RALGDS | NM_006266 | ral guanine nucleotide dissociation stimulator |
| RANBP17 | NM_022897 | RAN binding protein 17 |
| RANBP2 | NM_006267 | RAN binding protein 2 |
| RAP1GDS1 | NM_021159 | RAP1, GTP-GDP dissociation stimulator 1 |
| RARA | NM_001145302 | retinoic acid receptor, alpha |
| RBM14 | NM_006328 | RNA binding motif protein 14 |
| RBM15 | NM_022768 | RNA binding motif protein 15 |
| RBMS1 | NM_016836 | RNA binding motif, single stranded interacting protein 1 |
| RBPMS | NM_006867 | RNA binding protein with multiple splicing |
| REL | NM_002908 | v-rel reticuloendotheliosis viral oncogene homolog (avian) |
| RET | NM_020975 | ret proto-oncogene |
| RGS22 | NM_015668 | regulator of G-protein signaling 22 |
| RHOH | NM_004310 | ras homolog family member H |
| RMI2 | NM_152308 | RMI2, RecQ mediated genome instability 2, homolog (S. cerevisiae) |
| RNF130 | NM_018434 | ring finger protein 130 |
| RNF213 | NM_020954 | ring finger protein 213 |
| RNF216 | NM_207116 | ring finger protein 216 |
| ROS1 | NM_002944 | c-ros oncogene 1 , receptor tyrosine kinase |
| RPL22 | NM_000983 | ribosomal protein L22 |
| RPL5 | NM_000969 | ribosomal protein L5 |
| RPN1 | NM_002950 | ribophorin I |
| RPS2P32 | NR_026676 | ribosomal protein S2 pseudogene 32 |
| RRP15 | NM_016052 | ribosomal RNA processing 15 homolog (S. cerevisiae) |
| RSPO2 | NM_178565 | R-spondin 2 |
| RSPO3 | NM_032784 | R-spondin 3 |
| RUNX1 | NM_001754 | runt-related transcription factor 1 |
| RUNX1T1 | NM_175636 | runt-related transcription factor 1; translocated to, 1 (cyclin D-related) |
| RUNX2 | NM_004348 | runt-related transcription factor 2 |
| SDC4 | NM_002999 | syndecan 4 |
| SEC16A | NM_014866 | SEC16 homolog A (S. cerevisiae) |
| SEC31A | NM_016211 | SEC31 homolog A (S. cerevisiae) |
| SEPT5 | NM_002688 | septin 5 |
| SEPT6 | NM_145802 | septin 6 |
| SEPT8 | NM_015146 | septin 8 |
| SEPT9 | NM_006640 | septin 9 |
| SET | NM_003011 | SET nuclear oncogene |
| SETBP1 | NM_015559 | SET binding protein 1 |
| SFPQ | NM_005066 | splicing factor proline/glutamine-rich |
| SH3GL1 | NM_003025 | SH3-domain GRB2-like 1 |
| SLC1A2 | NM_004171 | solute carrier family 1 (glial high affinity glutamate transporter), member 2 |
| SLC22A1 | NM_153187 | solute carrier family 22 (organic cation transporter), member 1 |
| SLC26A6 | NM_134426 | solute carrier family 26, member 6 |
| SLC34A2 | NM_006424 | solute carrier family 34 (sodium phosphate), member 2 |
| SLC45A3 | NM_033102 | solute carrier family 45, member 3 |
| SMARCA5 | NM_003601 | SWI/SNF related, matrix associated, actin dependent regulator of chromatin, subfamily a, member 5 |
| SMO | NM_005631 | smoothened, frizzled family receptor |
| SND1 | NM_014390 | staphylococcal nuclease and tudor domain containing 1 |
| SNX29 | NM_032167 | sorting nexin 29 |
| SOX2 | NM_003106 | SRY (sex determining region Y)-box 2 |
| SP3 | NM_003111 | Sp3 transcription factor |
| SPECC1 | NM_152904 | sperm antigen with calponin homology and coiled-coil domains 1 |
| SPECC1L | NM_015330 | sperm antigen with calponin homology and coiled-coil domains 1-like |
| SQSTM1 | NM_003900 | sequestosome 1 |
| SRC | NM_198291 | v-src sarcoma (Schmidt-Ruppin A-2) viral oncogene homolog (avian) |
| SRGAP3 | NM_014850 | SLIT-ROBO Rho GTPase activating protein 3 |
| SRMS | NM_080823 | src-related kinase lacking C-terminal regulatory tyrosine and N-terminal myristylation sites |
| SRSF3 | NR_036610 | serine/arginine-rich splicing factor 3 |
| SS18 | NM_005637 | synovial sarcoma translocation, chromosome 18 |
| SS18L1 | NM_198935 | synovial sarcoma translocation gene on chromosome 18-like 1 |
| SSBP2 | NM_012446 | single-stranded DNA binding protein 2 |
| SSH2 | NM_033389 | slingshot homolog 2 (Drosophila) |
| SSX1 | NM_005635 | synovial sarcoma, X breakpoint 1 |
| STAT3 | NM_213662 | signal transducer and activator of transcription 3 (acute-phase response factor) |
| STAT5B | NM_012448 | signal transducer and activator of transcription 5B |
| STAT6 | NR_033659 | signal transducer and activator of transcription 6, interleukin-4 induced |
| STIL | NM_003035 | SCL/TAL1 interrupting locus |
| STL | NR_026876 | . |
| STRN | NM_003162 | striatin, calmodulin binding protein |
| SUSD1 | NM_022486 | sushi domain containing 1 |
| SUZ12 | NM_015355 | suppressor of zeste 12 homolog (Drosophila) |
| SVOPL | NM_174959 | SVOP-like |
| SYCP1 | NM_003176 | synaptonemal complex protein 1 |
| SYK | NM_003177 | spleen tyrosine kinase |
| TACC1 | NM_006283 | transforming, acidic coiled-coil containing protein 1 |
| TACC2 | NM_206862 | transforming, acidic coiled-coil containing protein 2 |
| TACC3 | NM_006342 | transforming, acidic coiled-coil containing protein 3 |
| TADA2A | NM_133439 | transcriptional adaptor 2A |
| TAF15 | NM_139215 | TAF15 RNA polymerase II, TATA box binding protein (TBP)-associated factor, 68kDa |
| TAF3 | NM_031923 | TAF3 RNA polymerase II, TATA box binding protein (TBP)-associated factor, 140kDa |
| TAL1 | NM_003189 | T-cell acute lymphocytic leukemia 1 |
| TAL2 | NM_005421 | T-cell acute lymphocytic leukemia 2 |
| TBCEL | NM_152715 | tubulin folding cofactor E-like |
| TBL1XR1 | NM_024665 | transducin (beta)-like 1 X-linked receptor 1 |
| TCEA1 | NM_201437 | transcription elongation factor A (SII), 1 |
| TCF12 | NM_207040 | transcription factor 12 |
| TCF3 | NM_003200 | transcription factor 3 (E2A immunoglobulin enhancer binding factors E12/E47) |
| TCF7L2 | NM_030756 | transcription factor 7-like 2 (T-cell specific, HMG-box) |
| TCL1A | NM_021966 | T-cell leukemia/lymphoma 1A |
| TCL6 | NR_028288 | T-cell leukemia/lymphoma 6 (non-protein coding) |
| TEC | NM_003215 | tec protein tyrosine kinase |
| TECTA | NM_005422 | tectorin alpha |
| TEK | NM_000459 | TEK tyrosine kinase, endothelial |
| TERT | NM_198253 | telomerase reverse transcriptase |
| TET1 | NM_030625 | tet methylcytosine dioxygenase 1 |
| TET2 | NM_017628 | tet methylcytosine dioxygenase 2 |
| TFE3 | NM_006521 | transcription factor binding to IGHM enhancer 3 |
| TFEB | NM_007162 | transcription factor EB |
| TFG | NM_006070 | TRK-fused gene |
| TFPT | NM_013342 | TCF3 (E2A) fusion partner (in childhood Leukemia) |
| TFRC | NM_003234 | transferrin receptor (p90, CD71) |
| TG | NM_003235 | thyroglobulin |
| THADA | NM_022065 | thyroid adenoma associated |
| TLX1 | NM_005521 | T-cell leukemia homeobox 1 |
| TLX3 | NM_021025 | T-cell leukemia homeobox 3 |
| TMCC1 | NR_033361 | transmembrane and coiled-coil domain family 1 |
| TMPRSS2 | NM_005656 | transmembrane protease, serine 2 |
| TNFRSF11A | NM_003839 | tumor necrosis factor receptor superfamily, member 11a, NFKB activator |
| TNFRSF17 | NM_001192 | tumor necrosis factor receptor superfamily, member 17 |
| TNFSF11 | NM_033012 | tumor necrosis factor (ligand) superfamily, member 11 |
| TOP1 | NM_003286 | topoisomerase (DNA) I |
| TP63 | NM_003722 | tumor protein p63 |
| TPM3 | NM_153649 | tropomyosin 3 |
| TPR | NM_003292 | translocated promoter region, nuclear basket protein |
| TRAF1 | NM_005658 | TNF receptor-associated factor 1 |
| TRIM24 | NM_015905 | tripartite motif containing 24 |
| TRIM27 | NM_006510 | tripartite motif containing 27 |
| TRIM33 | NM_033020 | tripartite motif containing 33 |
| TRIP11 | NM_004239 | thyroid hormone receptor interactor 11 |
| TSHR | NM_001142626 | thyroid stimulating hormone receptor |
| TTL | NR_024507 | tubulin tyrosine ligase |
| TYK2 | NM_003331 | tyrosine kinase 2 |
| UACA | NM_018003 | uveal autoantigen with coiled-coil domains and ankyrin repeats |
| UBE2L3 | NR_046082 | ubiquitin-conjugating enzyme E2L 3 |
| USH1G | NM_173477 | Usher syndrome 1G (autosomal recessive) |
| USP6 | NM_004505 | ubiquitin specific peptidase 6 (Tre-2 oncogene) |
| VAV1 | NM_005428 | vav 1 guanine nucleotide exchange factor |
| VCL | NM_014000 | vinculin |
| VTI1A | NM_145206 | vesicle transport through interaction with t-SNAREs homolog 1A (yeast) |
| WHSC1 | NM_133335 | Wolf-Hirschhorn syndrome candidate 1 |
| WHSC1L1 | NM_023034 | Wolf-Hirschhorn syndrome candidate 1-like 1 |
| WIF1 | NM_007191 | WNT inhibitory factor 1 |
| WT1 | NM_024426 | Wilms tumor 1 |
| WWTR1 | NM_015472 | WW domain containing transcription regulator 1 |
| XPO1 | NM_003400 | exportin 1 (CRM1 homolog, yeast) |
| YAP1 | NM_006106 | Yes-associated protein 1 |
| YES1 | NM_005433 | v-yes-1 Yamaguchi sarcoma viral oncogene homolog 1 |
| YPEL5 | NM_016061 | yippee-like 5 (Drosophila) |
| YWHAE | NR_024058 | tyrosine 3-monooxygenase/tryptophan 5-monooxygenase activation protein, epsilon polypeptide |
| YY1 | NM_003403 | YY1 transcription factor |
| ZBTB16 | NM_006006 | zinc finger and BTB domain containing 16 |
| ZC3H7B | NM_017590 | zinc finger CCCH-type containing 7B |
| ZMYM2 | NM_197968 | zinc finger, MYM-type 2 |
| ZNF384 | NM_133476 | zinc finger protein 384 |
| ZNF444 | NM_018337 | zinc finger protein 444 |
| ZNF521 | NM_015461 | zinc finger protein 521 |
| ZNF700 | NM_144566 | zinc finger protein 700 |
| ZNF703 | NM_025069 | zinc finger protein 703 |

**Table S4.** RIN values, decay rates and $\boldsymbol{R}^{\boldsymbol{2}}$ and p values for the model $\log\left( median coverage \right)=decay rate/1000\times distance+offset$, where median coverage is only calculated for expressed genes in the fusion list, for normal and tumor tissue samples with different levels of RNA degradation. Sample type (either tissue or cell line), histology and anatomical site is provided**.**

| **Type** | **Anatomical site** | **Histology** | **RIN** | **Decay rate** | $\boldsymbol{R}^{\boldsymbol{2}}$ | **p value** |
| --- | --- | --- | --- | --- | --- | --- |
| Tissue | Kidney | Normal | 3.4 | -0.74 | 0.87 | 3.78E-22 |
| Tissue | Lung | Adenocarcinoma | 6.4 | -0.76 | 0.94 | 3.99E-30 |
| Tissue | Lung | Adenocarcinoma | 7.7 | -0.76 | 0.95 | 1.83E-31 |
| Tissue | Lung | Metastatic cholangiocarcinoma | 7.7 | -0.62 | 0.94 | 6.10E-30 |
| Tissue | Liver | Normal | 7.8 | -0.22 | 0.95 | 4.13E-31 |
| Tissue | Colon | Normal | 8.2 | -0.67 | 0.97 | 1.71E-38 |
| Tissue | Liver | Normal | 8.3 | -0.39 | 0.99 | 4.97E-44 |
| Tissue | Lymph node | Normal | 8.4 | -0.18 | 0.97 | 7.42E-36 |
| Tissue | Peripheral blood | Acute progranulocytic leukemia (APL) | 8.5 | -0.11 | 0.86 | 2.74E-21 |
| Tissue | Lung | Adenocarcinoma | 8.5 | -0.15 | 0.90 | 1.09E-24 |
| Tissue | Lung | Adenocarcinoma | 9.0 | -0.07 | 0.68 | 8.23E-13 |
| Tissue | Uterus | Normal | 9.2 | -0.12 | 0.91 | 3.21E-26 |
| Tissue | Lung | Invasive grade 3 adenocarcinoma | 9.3 | -0.16 | 0.94 | 3.15E-29 |
| Cell line | Cell culture | Endometrial Stromal Nodule | 9.7 | -0.02 | 0.09 | 3.85E-02 |
| Cell line | Cell culture | Enchondroma v. fibromyxoid sarcoma | 9.9 | -0.03 | 0.11 | 2.27E-02 |
| Cell line | Cell culture | Ewings sarcoma | 10.0 | -0.01 | 0.06 | 1.00E-01 |
| Cell line | Cell culture | Liposarcoma | 10.0 | -0.02 | 0.09 | 3.39E-02 |
| Cell line | Cell culture | Anaplastic large cell lymphoma | 10.0 | -0.03 | 0.27 | 1.76E-04 |
| Cell line | Cell culture | Synovial Sarcoma | 10.0 | 0.01 | 0.01 | 4.23E-01 |
| Cell line | Cell culture | Extraskeletal myxoid chondrosarcoma | 10.0 | 0.01 | 0.02 | 3.25E-01 |

**Table S5.** Number of fusion supporting reads per 50 million reads for different fusions occurring in UHR at different levels of RNA degradation. The distance of the breakpoint from the 3’ end is also provided.

| **Fusion** | **Distance of gene fusion breakpoint from 3’ end of message (kb)** | **Number of gene fusion supporting reads by RIN value of UHR sample** | | | | | |
| --- | --- | --- | --- | --- | --- | --- | --- |
|  |  | **8.6** | **8.4** | **7.6** | **5.9** | **4.9** | **3.9** |
| *FOXA1-TTC6* | 0.7 | 22 | 34 | 29 | 51 | 47 | 28 |
| *BCAS3-BCAS4* | 1.0 | 84 | 109 | 113 | 122 | 109 | 96 |
| *GAS6-RASA3* | 1.8 | 28 | 22 | 21 | 11 | 8 | 10 |
| *ARHGEF2-SULF2* | 3.1 | 12 | 6 | 4 | 4 | 2 | 1 |
| *BCR-ABL1* | 5.3 | 15 | 5 | 3 | 1 | 0 | 1 |

**Table S6.** List of fusions detected in tumor samples from Supp. Table 3. The sample type, histologic diagnosis, RIN value, distance of the gene fusion breakpoint from the 3’ end, estimated sensitivity for the corresponding distance from the 3’ end to the breakpoint (calculated as in Fig. 3a) are also provided. For fusions whose distance is over 5kb, we included the estimated sensitivity at 5kb and denoted with the symbol *.

| **Type** | **Histologic diagnosis** | **RIN** | **Fusion detected by RNA seq** | **Distance from 3’ end** | **Estimated sensitivity** |
| --- | --- | --- | --- | --- | --- |
| Tissue | Metastatic cholangiocarcinoma | 7.7 | *FGFR2-TACC3* | 741 | 93.6 |
| Cell line | Synovial Sarcoma | 10.0 | *SS18-SSX1* | 832 | 95.3 |
| Cell line | Liposarcoma | 10.0 | *FUS-DDIT3* | 1,078 | 96.4 |
| Tissue | Acute progranulocytic leukemia | 8.5 | *RARA-PML* | 1,496 | 94.4 |
| Tissue | Lung Adenocarcinoma | 7.7 | *KIF5B-RET* | 1,833 | 75.7 |
| Tissue | Lung adenocarcinoma | 9.3 | *EML4-ALK* | 2,141 | 91.2 |
| Cell line | Anaplastic large cell lymphoma | 10.0 | *NPM-ALK* | 2,141 | 94.9 |
| Tissue | Acute progranulocytic leukemia | 8.5 | *PML-RARA* | 2,627 | 93.4 |
| Cell line | Ewings Sarcoma | 10.0 | *EWSR1-FLI1* | 3,000 | 94.2 |
| Cell line | Endometrial stromal nodule | 9.7 | *JAZF1-SUZ12* | 3,934 | 94.2 |
| Cell line | Extraskeletal myxoid chondrosarcoma | 10.0 | *EWSR1-NR4A3* | 4,907 | 95.0 |
| Cell line | Kasumi cell line | 10.0 | *AML1-ETO* | 7,002 | 94.9* |
| Cell line | Enchondroma v. fibromyxoid sarcoma | 10.0 | *HGMA2-LPP* | 16,622 | 87.0* |
